# Oviposition behaviour of *Rhagoletis completa* on dead end host *Cydonia oblonga*

**DOI:** 10.1038/s41598-025-88677-y

**Published:** 2025-04-01

**Authors:** Sándor Kecskeméti, Anna Laura Erdei, Janka Simon, Balázs Kiss, Béla Péter Molnár

**Affiliations:** 1 Department of Chemical Ecology, Centre for Agricultural Research, Plant Protection Institute, HUN-REN, Budapest, Hungary; 2 Department of Plant Protection Biology, Swedish University of Agricultural Sciences, Alnarp, Sweden; 3 Department of Zoology, Centre for Agricultural Research, Plant Protection Institute, HUN-REN, Budapest, Hungary

**Keywords:** *Rhagoletis completa*, Dead-end host, Plant volatiles, EAD, EPD, Quince, Chemical ecology, Behavioural ecology

## Abstract

Host finding behaviour and host acceptance for oviposition is a rather complex process amongst insects; it is a major decision that will have a direct effect on the performance of offspring. It is not uncommon that herbivorous insects oviposit on unsuitable hosts. The level of insect-host specialisation correlates with the likelihood of mis-oviposition incidence; polyphagous herbivore insect species have a higher chance to lay eggs on unsuitable hosts compared to specialised monophagous ones. Over several seasons, large numbers of walnut husk fly adults were observed on the canopy and fruit of European quince. Males were engaging in behaviours like fighting, courting, mating, and guarding oviposition sites, while females copulated, oviposited, or rested. Oviposition marks were found on quince fruits, with unhatched eggs, chorions, and newly hatched maggots inside. Molecular identification confirmed the species as *Rhagoletis completa*. To investigate this unusual host interaction, electrophysiological recordings were conducted on the antennae and maxillary palpi of WHF adults using quince and walnut fruit and foliage volatiles. Behavioural experiments also explored any preference for quince. The results showed that WHF adults, especially females, exhibited a positive attraction to quince fruit volatiles, indicating that their peripheral detection of volatiles is not limited to host-specific compounds. Based on these findings, we conclude that WHF adults treated quince as a suitable host for oviposition in a natural condition.

## Introduction

The western walnut husk fly (*Rhagoletis completa*, Tephritidae WHF) is a specialised tephritid fruit fly native to southern and Central America^1^. It was first detected in Europe in Switzerland in 1988^2^ and since then, it has spread across Europe. As there are no impermeable geographical barriers across the continent it is hypothesised that WHF has not reached the limits of its potential distribution^3^. WHFs are univoltine, exhibit long diapause periods in the soil, and closely synchronise their emergence with that of the fruiting phenology of their hosts. Based on the diet breadth, WHF could be considered as a stenophagous insect^4^ meaning feeding on plants within one genus. According to Medic et al.^5^ ten plants have been identified as hosts for *R. completa*. English or Persian walnut (*Juglans regia*) is the most commercially important^6^, while black walnut (*Juglans nigra*) is the native host^7^ and is of lesser commercial importance. Furthermore *Juglans microcarpa*, *J. major, J. hirsuta*^8^, *J. hindii*, *J. californica*^6^, and *J. mollis* also reported as natural hosts (EPPO, CABI). WHF is considered as the most important pest in commercial walnut production, the economic impact of WHF on international trade is significant, with annual damages estimated at 79 million euros^3^. Females of WHF oviposit clusters of eggs under the epidermis of walnut fruits. The resulting larval feeding induces blackish necrotic lesions on the fruit surface. The affected fruits experience premature abscission due to accelerated senescence, and the kernels have overall reduced sensory qualities. Furthermore, the compromised fruits become more vulnerable to secondary infections, intensifying crop losses. Without the implementation of effective phytosanitary treatments, it is estimated that up to 80% of the walnut crop could be lost due to WHF infestation^3^.

There is limited knowledge about alternative hosts of WHF, which may serve as reservoirs outside the Juglans genus. Yee and Goughnour reported that WHF adults emerged from field-collected English hawthorn^9^. Boyce^6^ suggested that peach could serve as a host for WHF, particularly when late-ripening varieties are planted near walnut trees. No-choice laboratory oviposition assays have demonstrated that WHF females successfully laid eggs on pear, nectarine, apple, and various other plants, including pepper, potato, tomato, orange, tangerine, grapefruit, lemon, and plum^6,10^. Additionally, while females attempted oviposition on fig, grape, prickly pear, pecan, avocado, and pomegranate, these attempts were unsuccessful^6^. These observations indicate that, under specific conditions, WHF females can oviposit on plants other than walnut.

It may initially seem counterintuitive that a highly specialised insect like the WHF would oviposit on unsuitable plants. However, the oviposition preference of insects does not always align with selecting the optimal host for offspring survival. In a study conducted by Wiklund with the butterfly *Papilio machaon*, one of the most preferred plant species for oviposition was *Bifora radians* yet this plant was not suitable for larval development, as mortality reached 100%. Conversely, almost zero percent of females oviposited on *Pastinaca sativa*, yet larval mortality was one of the lowest amongst the tested umbelliferous plants^11^. The phenomenon of mis-ovipositing to an unsuitable host is not unknown even in specialised insect groups. The cecidomyiid fly *Dasineura marginemtorque* could not distinguish between the susceptible and resistant genotype of its host plant *Salix viminalis* when it comes to choosing the best oviposition site for its offspring. Similarly, some varieties of black current (*Ribes nigrum*) are resistant to the gall midge *Dasineura tetensis*, larval survival is extremely low on resistant varieties, yet females can not discriminate against it^12^.

A similar mis-oviposition phenomenon was detected in the year 2021 and 2022, where abundant adults of WHF on European quince trees were observed in Romhány, Hungary. Upon closer examination, these adults exhibited behaviours typical of their natural host plant, including courtship, territorial behaviour amongst males, infights between male conspecifics, foraging of adults and female oviposition. The examined fruits exhibited puncture marks, similar to oviposition marks left by WHF on walnut fruits. Under the epidermis of quince, clusters of eggs and freshly hatched maggots were present. Olfaction plays a crucial role in the host plant selection process for Rhagoletis species, where volatile compounds emitted from ripening fruits are key olfactory cues in locating and discriminating among potential host plants. Since behaviour displayed by sexes of WHFs suggested that quince was accepted as a host in this particular case, our first objective was to determine if peripheral organ receptors respond to specific quince volatiles. Furthermore to evaluate the choice preference between quince and walnut fruits by WHF specimens in bioassay experiments.

In this study we describe the phenomenon that WHF uses European quince (*Cydonia oblonga*) fruits as potential oviposition site, however larvae ceased development therefore acted as a dead-end hosts. Several experiments were conducted to examine if oviposition on quince was driven by olfactory cues. We have studied the volatile headspace of leafy branches and fruits of both plant species and assessed the similarities and discrepancies between the two volatile profiles. We have conducted electrophysiological experiments; such as gas chromatography coupled electroantennography (GC/FID-EAD) and electro palpigraphy (GC/FID-EPD) to uncover the possibly physiologically active volatiles of European quince that can be sensed by the olfactory receptors of WHF adults. Several behaviour experiments were conducted in order to observe the choice preference of WHF adults towards European quince, and evaluate if there is any bias towards it when paired against persian walnut. We have limited knowledge regarding behaviour and the olfactory sensitivity of tephritid species of the temperate climatic zones and restricted understanding of chemical cues driving host identification. A deeper scrutiny of this anomalous host choice could both benefit the general understanding of walnut husk fly behaviour and identification of viable attractants that could be the base of future lure development for monitoring and mass trapping applications.

## Material and methods

### Collection of plant and insect material

Plant and insect materials were collected from a solitaire, home gardening European quince (*Cydonia oblonga*) tree (approx. 30 years old tree with a ca. 4.5 m high and 6 m wide canopy), near Romhány, permitted by the private home owner (N 47.915056, E 19.257526, Nógrád county, Hungary). Botanical identification of collected plant material as *Cydonia oblonga* was verified by Tibor Bárány (Curator of Dendrological Collection, National Botanic Garden, Centre for Ecological Research, HUN-REN, H-2163 Vácrátót, Alkotmány str. 2–4.) and collected plant materials were stored at National Botanic Garden (H-2163 Vácrátót, Alkotmány str. 2–4.) (Collection id: co202110).

Quince fruits (20 pieces) with discolored, black dots, signs of earlier oviposition or with observed oviposition marks were collected and stored while 20 other fruits were labelled and left on the tree as a control. Collected quince fruits were subsequently stored in laboratory conditions (21 ± 1 °C, 50–60 RH%) and larval development was monitored on a weekly basis for two months compared to those fruits left on the tree.

For molecular study, WHF adults collected on the spot using insect collection aspirator were asphyxiated in 70% ethanol and later morphologically identified according to Bush^1^ and Foote^13^.

For comparative analyses of *Cydonia oblonga* and *Juglans regia*, the collection of English walnut samples were permitted by the National Botanic Garden, botanical identification was done by Tibor Bárány. Collected specimens were stored at National Botanic Garden (Collection id: jr202209).

### Behavioural observation of R. completa specimens on quince fruit

**Fig. 1:**
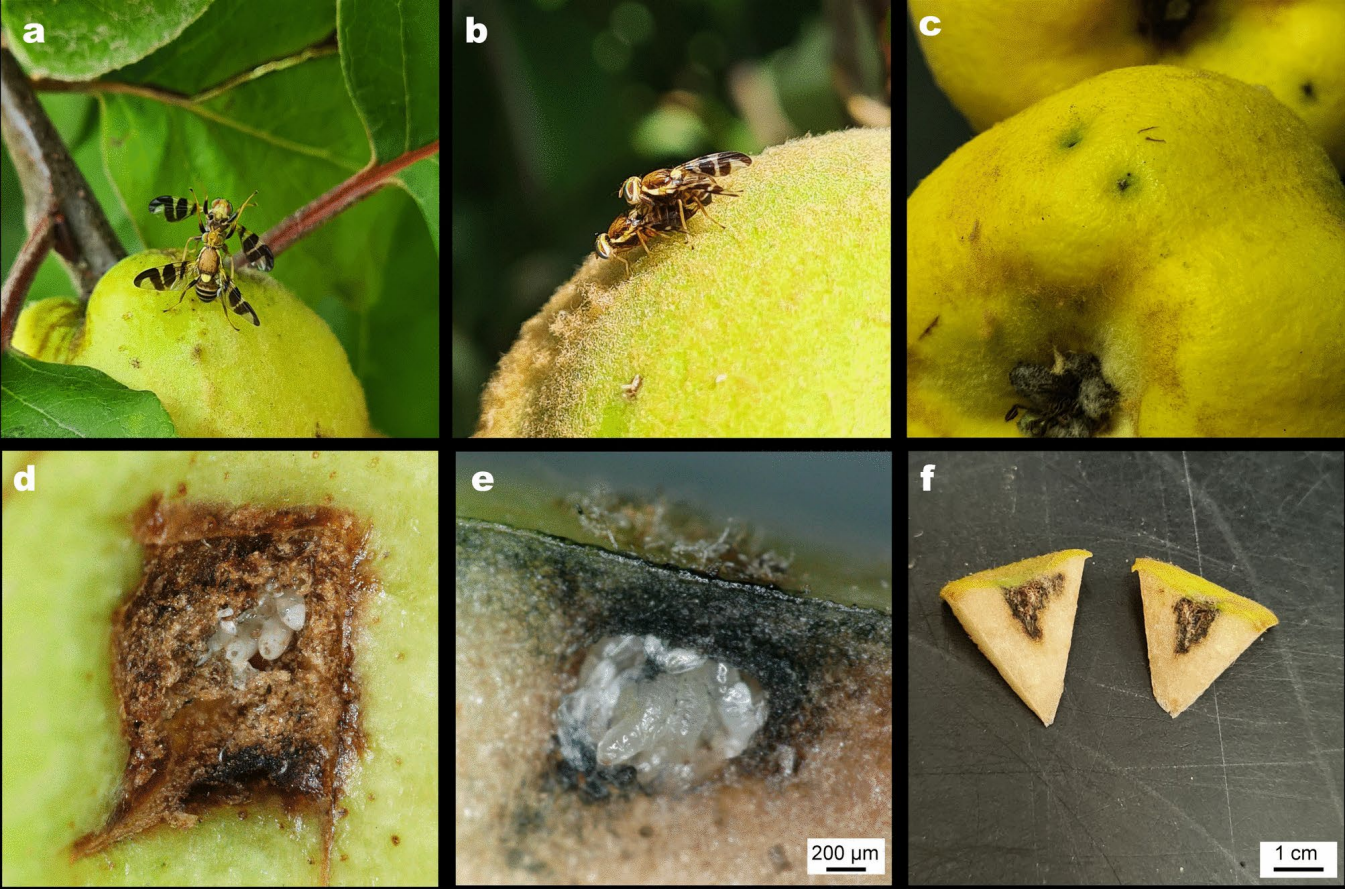
(**a**) Fight of the conspecific males of *Rhagoletis completa* on a quince fruit (**b**) mating pair of *Rhagoletis completa* on the surface of a quince fruit (**c**) oviposition marks on quince fruit caused by oviposition of female *Rhagoletis completa* (**d**) an open egg chamber with the freshly hatched larvae (**e**) cross section of egg chamber with the L2 larvae feeding on their egg shells (chorions) as well as on quince tissue (**f**) dissection of the affected fruit tissue reveal brown necrosis resulted from larval feeding.

Field observation of male fighting (Fig. 1a), courtship and mating (Fig. 1b) as well as female laying eggs on that particular quince tree was first made in early September of 2021 and subsequently was monitored on a weekly basis until late October. Male flies guarding the oviposition sites on the fruits after oviposition were also observed. Adult flies were also found on walnut seed-trees in the vicinity of the WHF affected quince tree, however in lower numbers compared to those found on the quince tree. The same phenomenon was observed in 2022, in the same months. Fruits with epidermal scars were examined with standard stereo microscopy and the epidermis layer was removed with a scalpel to verify insect activity in laboratory conditions. Some scar marks were removed alongside with neighboring tissue from the fruit for further dissection. All of our findings were photo-documented using a digital microscope (Keyence VHX-5000, Keyence Corporation, Osaka, Japan).

### Volatile collections and chemical analyses (GC–MS)

Headspace sampling in three replicates were made, using a cluster of 3 fruits at a time (BBCH 79 means that 90% of all fruit have a final size^14^) and young shoots with leaves from English walnut (*Juglans regia*) as well from quince (*Cydonia oblong*a) (BBCH 85, advanced ripening or fruit colouration stage) were performed. Cut branches and groups of fruits were isolated in Hewa roasting bags (35 cm × 43 cm, Hewa GmbH, Germany). The headspace was saturated for 1 h prior to sampling. Bags were equipped with a charcoal air filter inlet (30 g) while adsorbent tubes filled with 50 mg of HaySep Q (80–100 mesh) were connected on the opposite side using PTFE tubes and the headspace was sampled for 2 h. The adsorbed volatiles were eluted with 300 µl of n-hexane (purity 99.9%, VWR Chemicals) and kept at −40 °C. The extracts were subsequently used for electrophysiological recordings (GC-FID/EAD/EPD) and tentative chemical identification (GC–MS).

The plant volatiles were subsequently analysed using gas chromatography combined with mass spectrometry (GC–MS) (HP Agilent 5890 GC and 5973 MS, Agilent Technologies). Samples were analysed on standard non-polar capillary columns, HP-5 UI (30 m × 0.25 mm × 0.25 μm, J&W). The injector temperature was set to 250 °C and operated in splitless mode for 0.5 min. Oven temperature was maintained at 50 °C for 1 min, then increased at 10 °C min^−1^ to 280 °C and held for 4 min. The flow rate of the helium was 1.0 ml min^−1^. Positive electron ionisation (EI +) was used, with an electron energy level of 70 eV, 2 scans s^−1^ were recorded in the range of 35–400 m/z.

Compounds were tentatively identified by matching their mass spectra with those in the MS Libraries (NIST 23 and Wiley) using MassHunter (B.08.00, Agilent USA). Kováts retention indices were also calculated for all compounds using C8-C20 alkanes calibration standards. Calculated RIs were compared to RI values available in the NIST database. Furthermore, fragmentation pattern, retention time and retention indices were also compared with those of synthetic standards of high purity (> 98%) (Sigma Aldrich, etc.) when available (Supplementary Table 2). Identical volatile collection setups, comprising empty cooking bags and adsorbent filters, were additionally prepared as control systems. The volatile compounds detected in these control samples were subsequently subtracted from those identified in the plant headspace samples to ensure accuracy in the analysis.

### Electrophysiological recordings of R. completa (GC-FID/EAD and GC-FID/EPD)

To verify if the olfactory receptors can activate to non-host plant volatiles, multiple electrophysiological experiments were carried out on the antennae and maxillary palpi of WHF specimens. Identification of electrophysiologically active compounds in the volatile headspace of walnut and quince samples were carried out by gas chromatography coupled electroantennographic (GC-FID/EAD) and electropalpic detection (GC-FID/EPD). An Agilent 6890 N gas chromatograph (Agilent Technologies Inc., Santa Clara, CA, USA), equipped with an HP-5 capillary column (30 m × 0.32 mm × 0.25 μm, J&W Scientific, Folsom, CA, USA) and a flame ionisation detector (FID) was used for separations. From each volatile sample, 2 μl was injected into a 220 °C injector port in splitless mode. The initial oven temperature was held at 50 °C for 1 min, and increased by a steady rate of 10 °C min^−1^, to a maximum of 230 °C. The carrier Helium gas was maintained at a constant flow rate of 2 ml min^−1^. The GC effluent was split equally in a low dead volume glass four-way splitter. Two pieces of deactivated fused silica capillary columns (100 cm × 0.32 mm × 0.25 μm) were connected to the four-way splitter; one led to the flame ionisation detector (FID) (280 °C) and the other led to a heated (240 °C) EAD transfer line (Syntech, Kirchzarten, Germany) and into a glass tube (10 mm I.D.) with a charcoal-filtered and humidified airflow of 1 l min^-1^ that was led over to the antennal preparation.

For electrophysiological recordings WHF adults were fitted inside a pipette tip (100–1000 μl). The apex part of the pipette was cut off (~ 3–4 mm) so that only the heads of adults were able to protrude out of the pipette tip. In order to secure the insect inside the pipette, a cotton ball was gently pushed against its abdomen. The pipette was then positioned in front of the humidified airflow. For recordings, two glass capillary filled with Ringer solution^15^ were fixed on silver electrodes and connected to a pre-amplifier, (amplification strength of 10 folds) (EAG Combi Probe, Ockenfels Syntech GmbH, Germany) and recorded signals were converted into digital (IDAC-2, Ockenfels Syntech). For EAD measurements the reference capillary was pierced into the *occiput* region of the head, while the recording capillary touched the distal end of the antenna. During EPD readings, the reference capillary pierced the left compound eye, while the recording capillary touched the distal tip of a single maxillary palpus. A third empty glass capillary was fixed on a micro manipulator, which helped to secure the palpi in place and eliminated any kind of movement of mouthparts during recordings.

Electrophysiological experiments (EAD/EPD) for both sexes were performed in triplicates for each plant sample via GC-EAD software (ver. 1.2.3. Syntech Inc, Germany). Only responses consistent across all replicates were analysed further. The fundamentals of data preparation and analysis was based on Biasazin et al.’s publication^16^. The raw data of each electrophysiological recording of a given plant sample was normalised by the average antennal response observed within the same trace. Furthermore, for the visual representation of data in heatmap, the normalised responses given to each compound were adjusted to a single area unit (%) of the corresponding compound (*adj.resp*). The three repetitions of an experimental trial were averaged (*avr.adj.resp*) and were transformed by the *log(1* + *avr.adj.resp)* function. This method of data transformation was uniformly applied to all EAD/EPD experiments.

### Y-tube behaviour bioassay

Bioassay experiments were performed in order to support our on-field observations, that WHF adults were interested in quince fruits, furthermore we were curious if quince would be preferred over walnuts. To do so, two-choice behaviour experiments were conducted. Experiments were performed in a Y-tube glass system. Constant air flow was supplied to the system by an air pump. The effluent air was filtered through activated charcoal (30 g), and humidified via a gas-bubbler filled with sterile distilled water. The filtered and humidified air was split into two lines, and each splitted line connected to a cooking bag (35 × 45 cm, Toppits, Germany). Plant samples were placed separately inside the cooking bags. The air flowing through the cooking bag, carrying the volatilome of samples, was connected to the two diverging ends of the Y-tube with a threaded glass fitting. To ensure equal flow through the two diverging branches of the Y-tube, adjustable flow meters were also implemented (the air-flow through each line was 0.5 l min^−1^). Every tubing used in the setup was made from teflon and every glass component was fitted with Duran ground joints. To encourage the movement of adults, we placed a LED light source (V-TAC G-series, 36W, 4320 lm, 4500 K) approximately 1 m behind the experimental setup. Light stimulus was used during each experiment while no other luminous source was present. Experiments were carried out at 21 °C (± 1 °C) with 50–55% relative humidity.

To compare the choice preference of WHF adults between the fruits of quince and walnut, we used the aforementioned experimental setup. We placed ~ 500 g (± 50 g) of uncut quince and walnut fruit samples separately inside the cooking bags and initiated airflow. We placed 10 unmated adults of WHF simultaneously inside a small glass tube (one end covered with wire mesh), which could be inserted to the starting position of the Y-tube. After switching the light stimulus on, we observed the movement and choice of unmated WHFs. Only those choices were taken into account, when the insect released from the starting position covered at least 85% of total distance. Both sexes of WHF were tested separately and the experiment was repeated 10 times (in total the choice of 10 × 10 female and 10 × 10 male WHFs were recorded). The maximum time for WHF adults to choose between samples was 5 min. We flipped the experimental setup by its longitudinal axis by 180° in every two repetition to eliminate positional effect. Non responding specimens were not excluded from data analyses. The Y-test tubes were washed with acetone (≥ 99.5% Sigma Aldrich) after every repetition/experimental trial, and heat sterilised at 200 °C for 1 h.

To assure the reliability of our experimental setup, we conducted a blank vs blank experiment with the same parameters described above, but with the absence of any test material. Specimens of male and female WHF adults were placed one by one in the experimental setup separately. In total the choice of 30 female and 30 male WHF adults were recorded. Non-responding specimens were excluded from this analysis.

### Molecular methods

Molecular examination was carried out to reinforce our morphological identification that the collected insects were *R. completa* adults. The insect samples collected from quince fruits were stored in 70% EtOH, and samples of *R. completa* maggots (collected from the husk of *Juglans regia*) were also used as reference for sequence analyses.

Total genomic and mtDNA was extracted from whole *Rhagoletis* specimens using Extraction Solution and Dilution Solution (Sigma Aldrich, Saint Louis, MO, USA) according to manufacturer’s protocol.

PCR products were sent for sequencing to LGC Genomics GmbH (Germany). Sequencing was done with the same primers which were used for amplifications. Resulting chromatograms were manually curated using MEGA11 software. Sequences were used as a query in a blastn search in GenBank. Best hit (most similar) sequences were collected, and were aligned with our newly determined sequences using MAFFT online with the G-INS-i algorithm (other settings were used as defaults). Leading and trailing gaps were coded as unknown characters. The alignment consisted of 18 sequences and 655 characters. *R. cingulata* was selected as an outgroup based on earlier results^17^.

Bayesian phylogenetic analysis was done following Glover et al.^17^, using MrBayes 3.1.2^18^. A maximum likelihood (ML) analysis was conducted with raxmlGUI 1.5^19,20^. Clade supports were calculated from 1000 bootstrap replicates. In both analyses, GTR + G was used as the nucleotide substitution model. Resulting phylogenetic trees were visualised with TreeGraph 2.13.0^21^.

### Data analysis

To evaluate the choice preference within male and female sexes of WHF adults between the fruits of european quince and persian walnut, we used one-way ANOVA model, with the fruit species considered as a fixed factor. Normality of residuals was verified by Shapiro–Wilk’s test. To reveal statistically homogeneous groups in attraction data in choice preference, Tukey’s post-hoc tests were performed, as Levene’s Test for equal variances was not violated.

To see whether the distribution of frequencies differs in male and female WHF adults in the no-choice experiment, we conducted chi-square goodness of fit tests with expected frequencies of 1:1 distribution.

ANOVA and chi-square test was performed via IBM SPSS Statistics ver. 22 (IBM Corporation, Armonk, New York, USA). Transformation of raw data was done in Microsoft Office Excel 2016 (Microsoft Corporation, Redmond Washington, USA). Heatmap visualization was created with R Studio ver. 2022.07.1 Build 554 (R Core Team, 2023) using the package “ggplot2”^22^. Additional image editing was done using Adobe Illustrator (Adobe Systems, Mountain View, California, USA).

## Results

### Behavioural observation

Adult tephritid flies, morphologically similar to WHF, were observed on a quince tree in Romhány, Hungary in high density. Several well characterised Tephritid behaviour steps including fights between conspecific males (Fig. 1a), courtship behaviour, mating (Fig. 1b), female egg laying as well as male guarding oviposition sites were observed and documented. All the tephritid specimens collected on the spot were morphologically identified as WHF (17 males, 15 females). Upon inspection of fruits, small, dark discolored dots (0.5–2 mm) were observed on the exocarp, which resembled puncture marks (Fig. 1c), similar to those of WHF seen on walnut husks. The marks were circular and the ovipositor insertion hole remained open (approx. 0.1 mm in diameter) and on the perimeter callus tissue started to form. Occasionally, oviposition sites also led to the deformation of the fruits. Underneath the stained area in the tissue cavity, eggs in clusters of 8–12 or maggots were found resembling tephritid larvae (Fig. 1d,e). The dissection of the affected fruit tissue revealed necrosis resulting from larval feeding (Fig. 1f, video supplement), however the observed damage on quince fruits was insignificant. In a set of quince fruits with puncture marks similar to those documented, larval development was arrested and aborted in the relatively early stages. In laboratory investigation of damaged fruits no older larvae than second instar were recovered alive from the fruit cavity. Subsequently only perished, shriveled larvae were found therefore no adult WHFs emerged from the examined quince fruits.

### Molecular methods

**Fig. 2:**
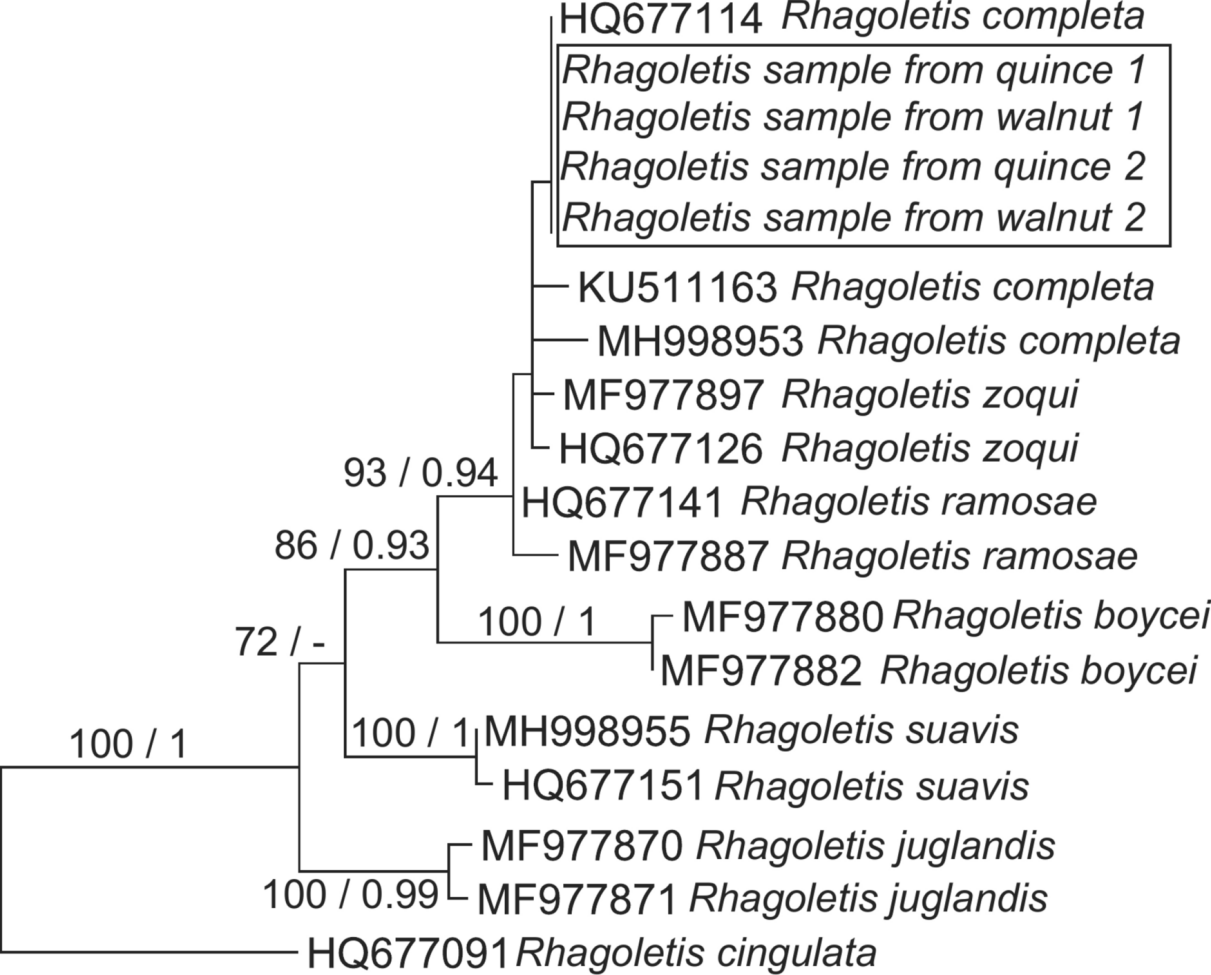
Highest likelihood phylogenetic tree from the maximum likelihood (ML) analysis Rhagoletis cytochrome oxidase subunit 1 (COI) gene sequences. Bootstrap support values calculated from 1000 replicates in ML analysis are shown on the branches (values below 70% are not shown), followed by posterior probabilities (not shown below 90). Bar indicates 0.01 expected changes per site per branch.

To complement morphological taxonomic identification, genetic analysis was completed as well. Four sequences were obtained from maggots isolated from either walnut or from quince fruits (Rhagoletis sample from quince 1 and 2; Rhagoletis sample from walnut 1 and 2), which were identical to each other (Fig. 2). Furthermore, a sequence determined to be *R. completa* in Switzerland (HQ67711) was also identical to the ones we obtained. Together with sequences observed from GenBank as *R. completa*, *R. zoqui* and *R. ramosae,* the four newly determined sequences from Hungarian samples formed a well supported (bootstrap support = 93; posterior probability = 0.94) clade (Fig. 2). Our resulting sequences have been uploaded to NCBI GeneBank database with accession number: OM392068, OM392069, OM392070 and OM392071.

### Volatile profile chemical analyses (GC–MS)

Gaschomatography coupled mass spectrometry analyses of walnut and quince fruit/foliage headspace revealed qualitative differences in the profile. Quince fruit compared to foliage was distinctly different. Dominant proportion of the compounds in fruit samples of quince were esters (~ 68%) while the second largest group consisted of terpenoids (~ 8%). From the detected compounds of quince foliage larger segments were, terpenoids (~ 29%), hydrocarbons (~ 21%), alkanes (~ 10%) and alcohols (~ 7%). Identified compounds between quince fruit and foliage were: ethyl isobutyrate; toluene; 2-hexanone; ethylbenzene; *p*-xylene; *o*-xylene; 1-ethyl-3-methylbenzene; *β*-myrcene; (*Z*)-3-hexenyl-acetate; hexyl acetate; *p*-cymene; limonene; nonanal; (*E*)-*β*-caryophyllene; *α*-humulene and *α*-farnesene.

The volatile profile of walnut fruit and foliage headspace samples shared a fair similarity. The number of detected peaks in walnut fruit was 96 compared to 107 in foliage. ~ 41% of compounds were terpenoids in fruit volatilome, compared to ~ 36% in foliage. In both samples, the second largest chemical group were hydrocarbons (fruit ~ 11%; foliage ~ 9%). Few esters were also detected in walnut fruit and foliage, around 10% of identified compounds in fruit and ~ 7% in foliage volatilome. The compounds that were detected and identified in walnut fruit but absent in foliage were: 1-methoxy-2-propyl acetate; heptanal; octanal; *α*-phellandrene; nonanal; decanal; ethyl 9-decenoate; and tetradecane, 2,6,10-trimethyl-.

Comparing the two fruit samples, toluene; 2-hexanone; butyl acetate; ethyl 2-methylbutyrate; ethyl isovalerate; ethylbenzene; *p*-xylene; 2-heptanone; *o*-xylene; *β*-myrcene; ethyl hexanoate; (*Z*)-3-hexenyl-acetate; *p*-cymene; limonene; nonanal; decanal; phenylethyl acetate; *β*-bourbonene; ethyl 9-decenoate; (*E*)-*β*-caryophyllene; (*Z*)-*β*-farnesene; *α*-humulene; *α*-farnesene were shared.

Across all four plant samples, the identified compounds that were shared: toluene; 2-hexanone; ethylbenzene; *p*-xylene; *o*-xylene; *β*-myrcene; (*Z*)-3-hexenyl-acetate; *p*-cymene; limonene; (*E*)-*β*-caryophyllene; *α*-farnesene.

### Electrophysiological recordings (GC-FID/EAD/EPD)

**Fig. 3:**
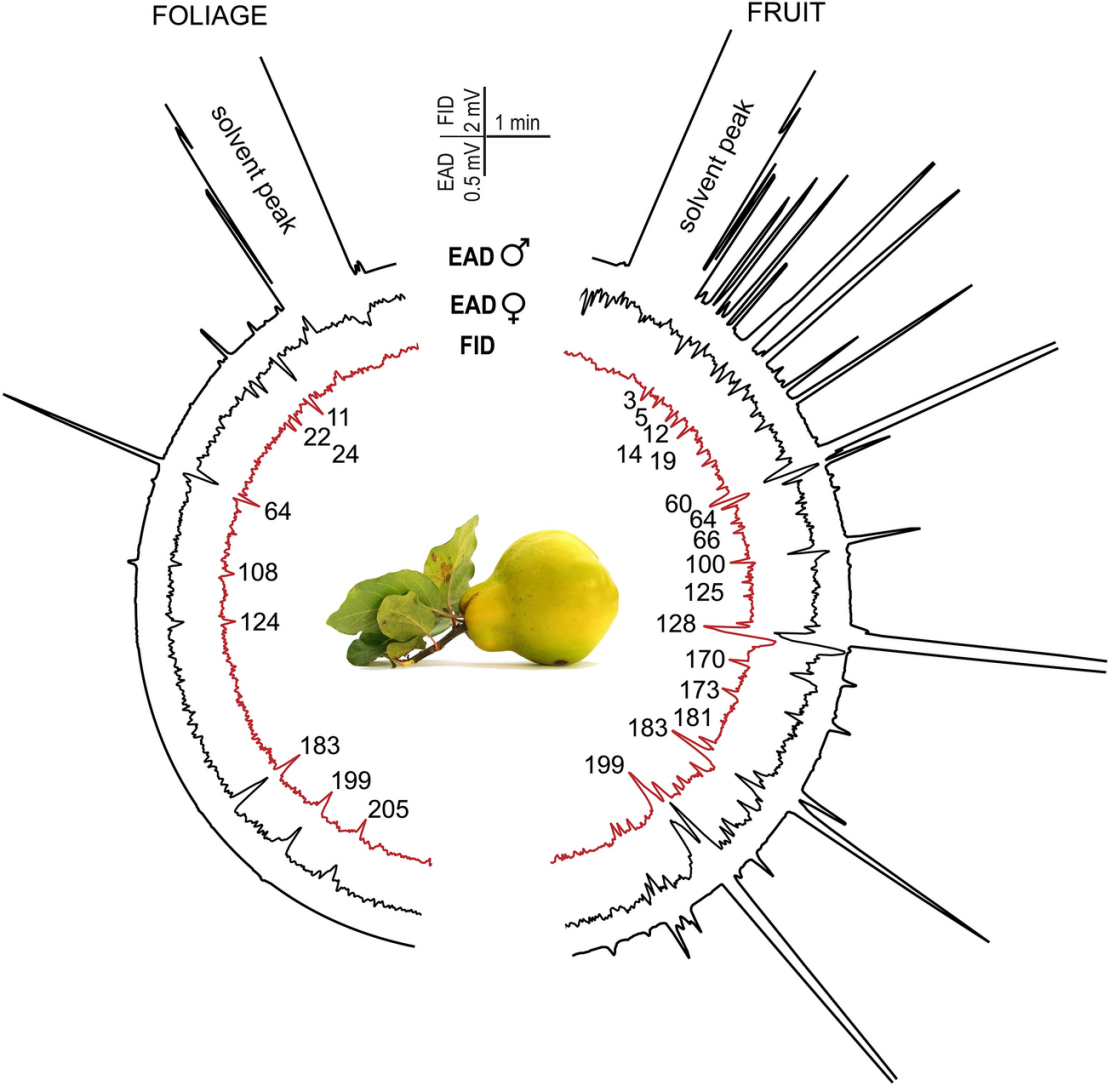
Representative electrophysiological responses from the tip of the antennae of female (black EAD traces) and male (red EAD traces) *Rhagoletis completa* to the volatile collection of *Cydonia oblonga* leaves (left semicircle) and quince fruit (right semicircle). As shown in Table S1. the numbers correspond to the compounds identified in the *Cydonia oblonga* volatile profile.

**Fig. 4:**
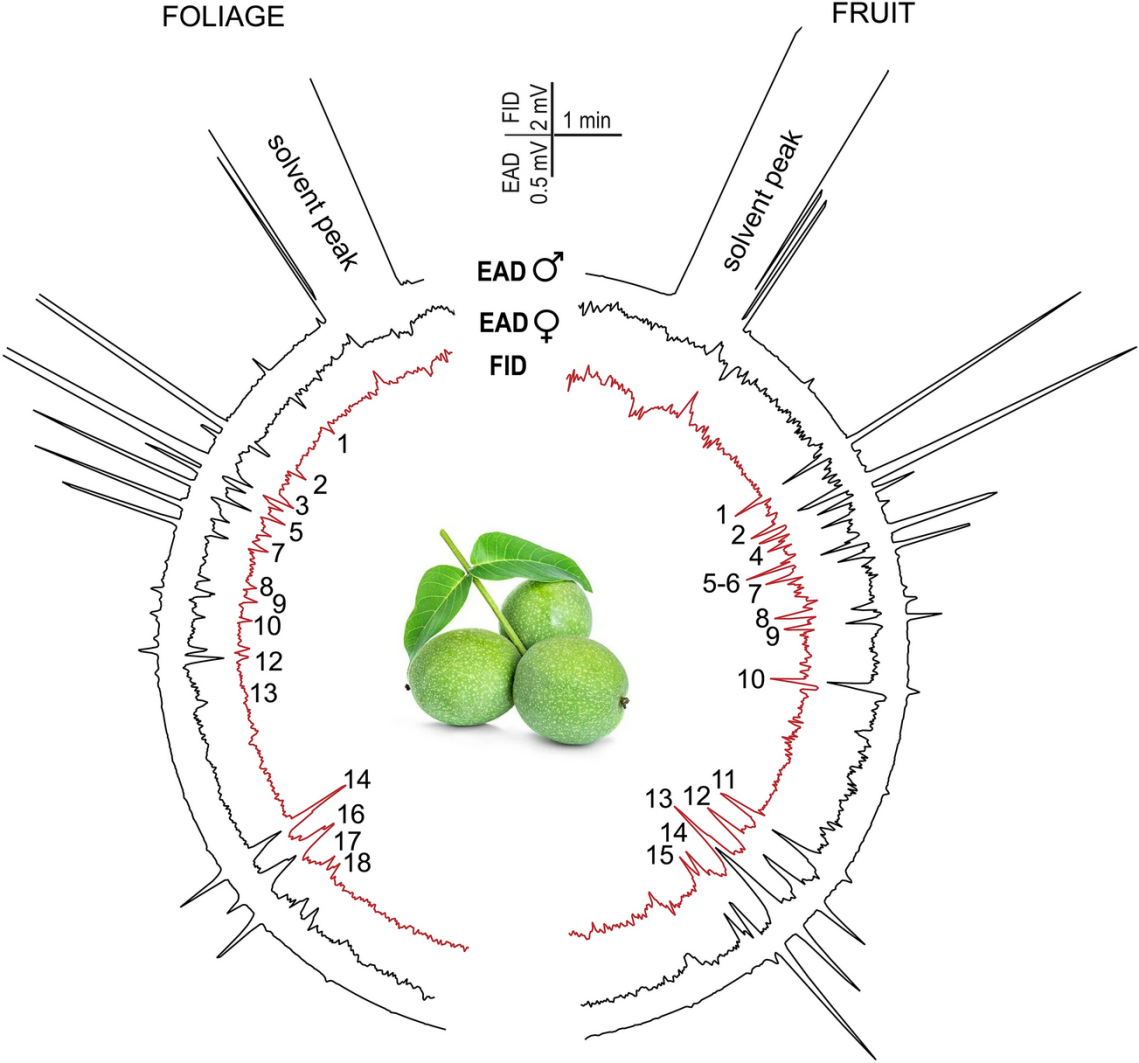
Representative electrophysiological responses from the tip of the antennae of female (black EAD traces) and male (red EAD traces) *Rhagoletis completa* to the volatile collection of walnut (*Juglans regia*) leaves (left semicircle) and quince fruit (right semicircle). Nine volatile compounds from the foliage and thirty from the fruit evoked antennal responses. As shown in Table S1. the numbers correspond to the compounds identified in the *Juglans regia* volatile profile.

**Fig. 5:**
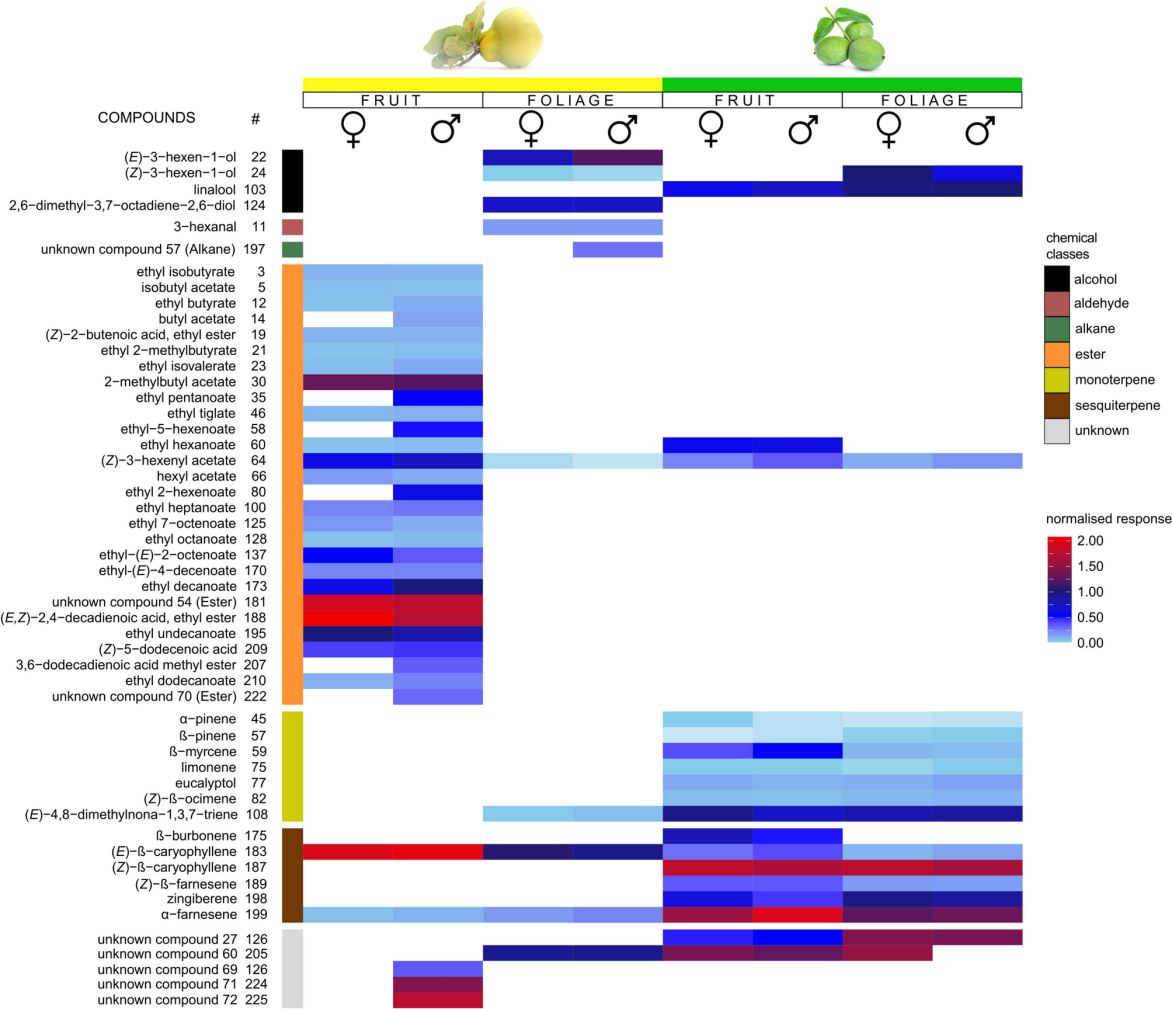
Heatmap illustrating antennal responses measured by GC-EAD using fruit and foliage headspace collection of *Cydonia oblonga* and *Juglans regia*. From left to right columns show names of the identified volatile constituents, ordinal numbers correspond with Table S1. summarized constituent numbers of the identified volatile compounds, color code for chemical classes, heatmaps of female and male *R. completa* antennal responses to *Cydonia oblonga* and *Juglans regia* fruit and foliage volatiles. Compounds are organized according to chemical classes and respective retention time.

Quince and walnut fruit and foliage volatilomes contained 225 compounds in total, 52 of which elicited consistent antennal responses (Fig. 3 and Fig. 4). Most of the physiologically active volatiles were esters and their derivatives (53%). Said compounds were almost exclusively unique to quince fruit, and only one compound, (*Z*)-3-hexenyl acetate was present in all examined plant parts (Fig. 5). Butyl acetate, ethyl-2 methylbutyrate and ethyl isovalerate were detected in walnut fruit and foliage samples, but in such low amounts, that these compounds did not elicit antennal response. The second largest chemical groups were monoterpenoids (7 compounds) and sesquiterpenoids (6 compounds). Most of the volatiles (76%) were only present in walnut samples. The compound (*E*)-4,8-dimethyl-1,3,7-nonatriene (DMNT) was also apparent in quince foliage. (*E*)-*β*-caryophyllene and α-farnesene existed in all four plant samples. We detected a discrepancy in the case of *ß*-bourbonene; as the relative amount in quince fruit was orders of magnitude larger compared to walnut fruit/foliage samples, yet it did not show to be physiologically active when quince fruit samples were tested during EAD recordings. The full list of antenally active volatiles is shown in (Fig. 5).

**Fig. 6:**
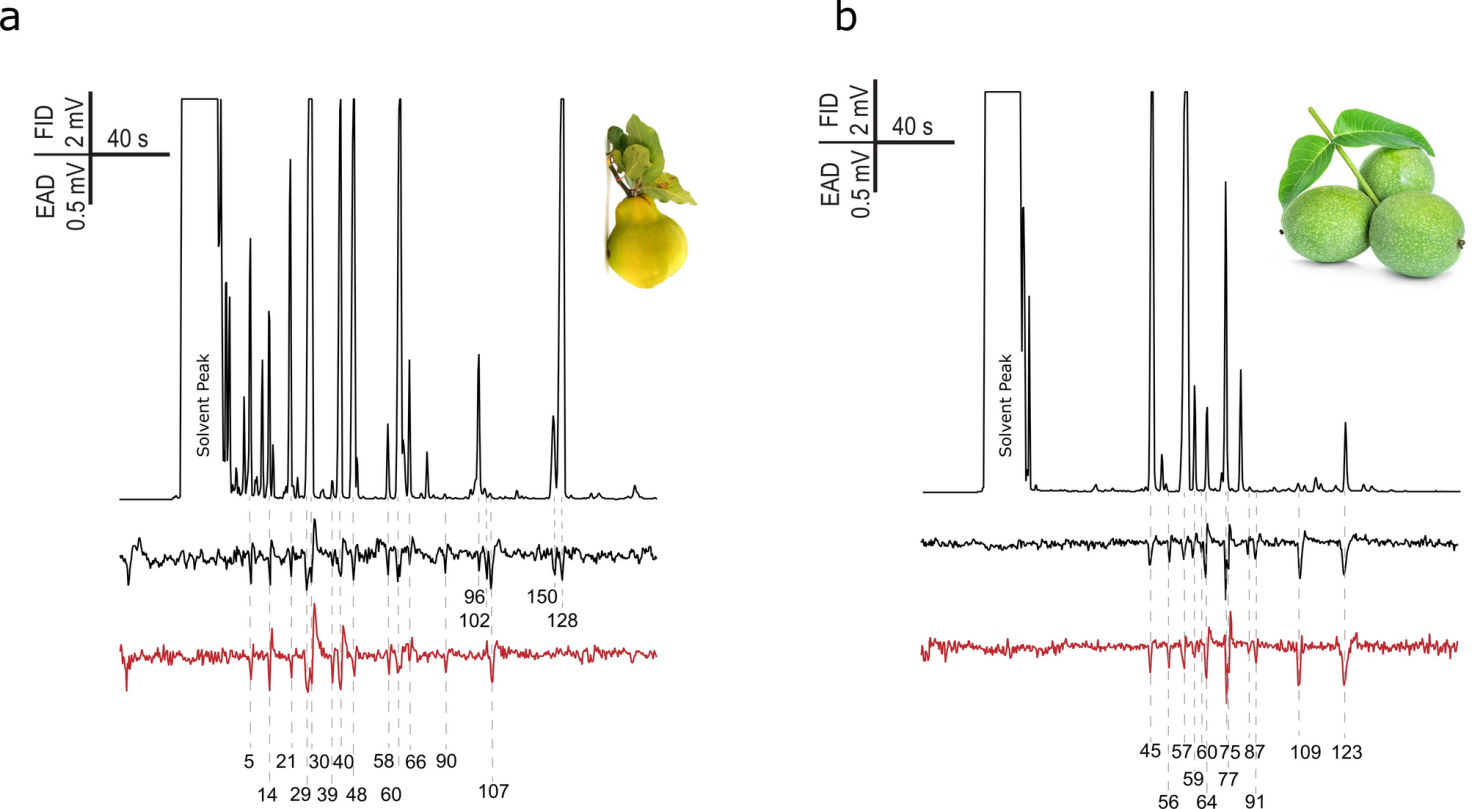
Electrophysiological responses from the palpus (GC-EPD) of female (black EDP traces) and male (red EPD traces) *Rhagoletis completa* adults to the volatile collections of (**a**) *Cydonia oblonga* fruits and (**b**) *Juglans regia* fruits. All together 17 volatile compounds from *Cydonia oblonga* fruit and 12 from *Juglans regia* fruit evoked responses from palpi. Mass-spectrometry identified compounds are included as a separate table (Table.S1).

**Fig. 7:**
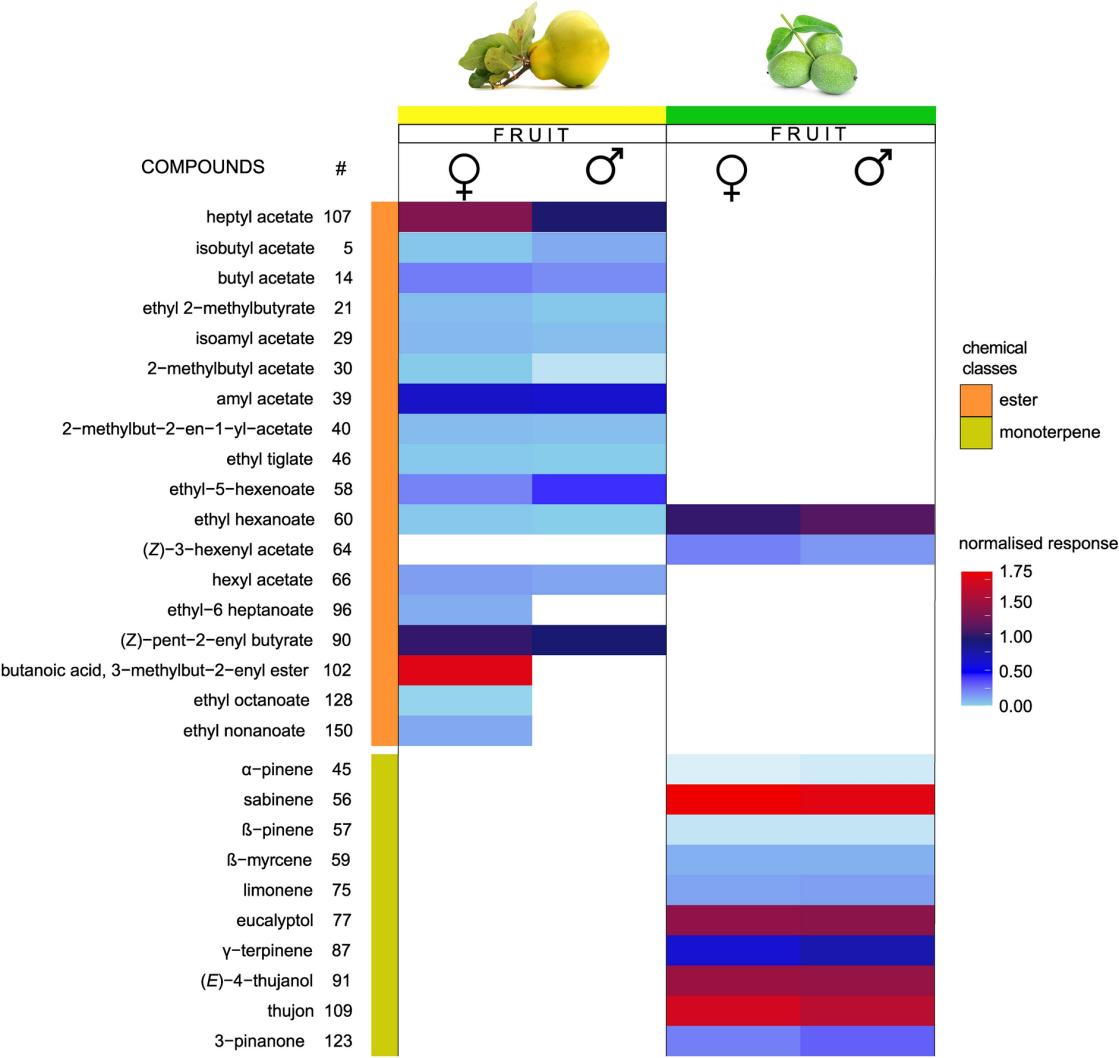
Heatmap illustrating palpi responses measured by GC-EPD using fruit headspace collection of *Cydonia oblonga* and *Juglans regia*. From left to right columns show names of the identified volatile constituents, ordinal numbers correspond with Table S1., summarized constituent numbers of the identified volatile compounds, chemical classes, heatmaps of female and male *R. completa* palpi responses to *Cydonia oblonga* and *Juglans regia* fruit volatiles. Compounds are organized according to chemical classes and respective retention time.

A similar pattern was found for the active compounds in palpi. The 28 electrophysiologically active volatiles (Fig. 6 and Fig. 7) separated to two well defined chemical groups: 17 were esters, from which 15 were unique to quince fruit samples. Ethyl hexanoate and (*Z*)-3-hexenyl acetate were also detected in walnut fruit samples; however interestingly (*Z*)-3-hexenyl acetate did not elicit palpi response from either sex of WHFs when a quince volatile sample was used. Ethyl-6 heptanoate, butanoic acid, 3-methylbut-2-enyl ester, ethyl octanoate and ethyl nonanoate compounds were only physiologically active for female specimens.

From all the volatiles that elicited responses from the palpi, ten compounds belonged to the group of monoterpenoids.

*ß*-myrcene and limonene were present in both samples from both plants, but possibly due to their low relative amount (Table S1), they elicited responses only in case of walnut headspace samples.

Foliage and fruit of *J. regia* have remarkably similar volatile profiles that show mainly qualitativedifferences (Figs. 4 and 5). The total number of compounds in foliage and fruit overlaps by 74%. The antennal responses to walnut volatiles overlapped completely between fruit and foliage in 84% of the cases. Antennae responded particularly well to the mono- and sesquiterpenoids that dominated both the foliage and the fruit volatile profile.

Analyses of the quince volatile pattern however painted a different picture. Fruit and foliage share only 9% of the identified compounds (Figs. 3 and 5). Fruit volatilome is dominated by esters, while foliage was more evenly distributed, with mono- and sesquiterpenoids and hydrocarbons being the most prevalent compounds. The male and female antennae responded dominantly to esters in quince samples, with males responding to more volatile components compared to females.

### Y-tube behaviour experiments

Multiple chi-square tests were performed to examine if there is any bias in the empty experimental setup itself in male and female specimens of WHFs. Based on chi-square test of goodness of fit, the expected distribution did not differ significantly from the observed distribution in neither case of male and females [females: X^2^(1,N = 30) = 0.0 p = 1; males: X^2^(1,N = 30) = 0.37 p = 0.85].

**Fig. 8:**
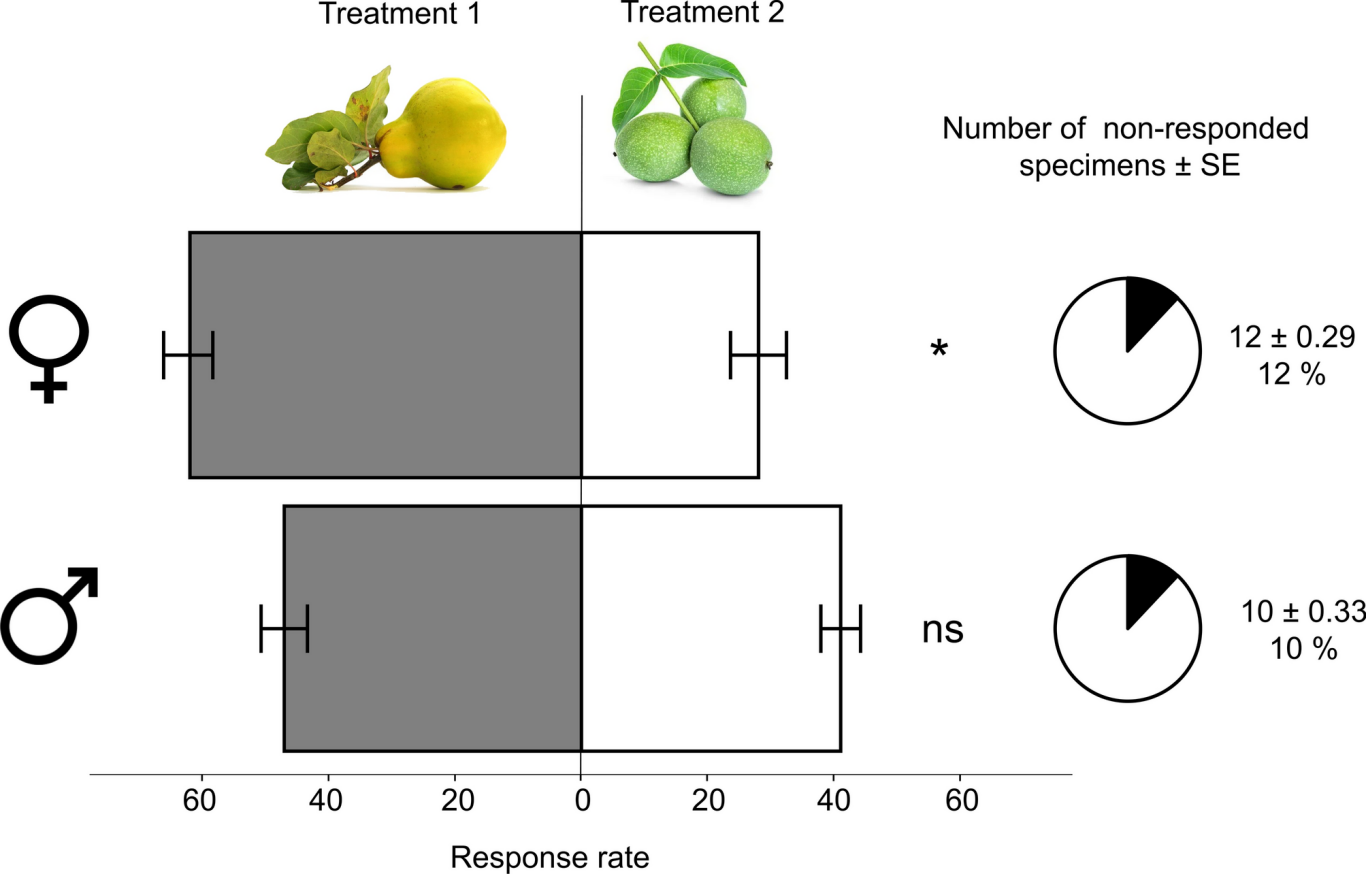
Fruit choice preference of unmated male and female WHF adults and the number of non responding specimens (± SE) in Y-tube bioassay. Asterisks indicate a significance between the homogenous groups revealed by Tukey’s post-hoc test (α = 0.05; p < 0.01).

However, when insects were compared against walnut and quince fruits, out of the 90 responding unmated female specimens, 62 significantly chose quince over walnut fruit (31%) (F(2.27) = 45.699 p < 0.001). ale respondents (n = 88) did not discriminate between fruit samples (F(2.27) = 33.073 p = 0.405) (Fig. 8).

## Discussion

The behaviour that WHF adults presented on quince trees may seem fundamental and trivial at first, but these actions incorporate host finding and oviposition behaviour. Mono- and stenophagous fruit fly species generally mate directly on oviposition substrate^4^ so naturally fruits are considered an invaluable ecological resource for fruit flies. We have recorded actively mating pairs on the fruit surface (Fig. 1b) of quince. It is exceptional, because Rhagoletis species (should) initiate copulation exclusively on their host plants ^23–26^. Furthermore we observed male conspecifics fighting (leg-kicking, boxing) on “oviposition resources”, which further suggests that quince fruits were important to WHF adults. The act of post copulatory mate guarding and defending oviposition sites increases fruit fly males’ fitness^8^, especially in the case of polygynandrous species like *Rhagoletis*^26,27^. The oviposition scars were guarded by males but not by females. Apart from mating on fruits, we noted copulation on the adaxial leaf surface as well, which is somewhat uncharacteristic for a specialised tephritid like *R. completa*. The marks left by females on fruit surfaces were distinct: Marks were circular, the centre did not fully close after ovipositor insertion, so marks had ~ 0,1 mm holes, which had callus tissue around the perimeter. These holes had blackish-dark brownish discolored halo (0.5–2 mm). Not always, but fruit tissue was also pitted/malformed. Under the fruit epidermis clutches of eggs (8–12) were recovered. The fact that WHF laid eggs inside quice is not insignificant. These specialised hostplant exploiters hardly make mistakes^12,28,29^, as these insects have a fewer number of ovarioles (as compared to polyphagous fruit flies)^30^, thus each egg has to be placed in the right place. Hostplant acceptance in Rhagoletis is a rather complex process; both visual and olfactory cues have to be “checked” in order to accept a plant as a host. For example *R. pomonella* relies solely on visual information alone to find the appropriate host if fruits are visually apparent, but use fruit odour together if fruits are unapparent^31^. Furthermore studies on several fruit fly species (including *R. completa*) showed that spherical fruit shape, fruit size and contrast to background is a dominant cue, when foraging for host plants^32–34^. Apart from visual appeal, host acceptance is also based on olfactory cues. Antennal olfactory receptors help guide the insect towards a suitable host plant by its volatiles. Surface chemicals are sampled by the specimen with tarsal and palpal contact chemosensory receptors, further analysing host suitability. Female specimens also have receptors on their aculeus, which signals vital information about the chemical composition of oviposition material^35^. All of these are key signals in host plant recognition and discrepancy sensed by the insect could result in the refusal of oviposition. Which means—in the case of quince—that either many (or all) of the requirements were fulfilled for WHF adults; or many male and female specimens consistently made the wrong choice leading to lethal consequences for their offspring.

The oviposition preference of insects should strongly correlate to the host suitability of their offsprings, since the maximisation of female fitness should be through the selection of high quality hosts for the next generation^36^. However this correlation ranges from excellent to poor in herbivorous insects^29^. The large number of “poor oviposition incidents” reported in literature^37^ has led to the formation of the “Optimal bad motherhood” principle, which predicts that females may prefer to oviposit on hosts that increase their own longevity. Sheirs et al.^38^ showed for the first time that the performance effect of a host plant on an adult herbivore will influence their oviposition decision. In the context of WHF and quince, it is possible that quince was perceived as a superior nutritional resource for WHF adults compared to walnuts.

The positive bias towards quince could be resolved with the examination and comparison of the volatile profiles of European quince and Persian walnut. One of the most striking differences is that overall, these volatile profiles couldn’t be further apart. Only a handful of antenally active compounds were shared in the two fruits. From the MS analyses, we can generally say that the most predominant compounds in walnut fruit profile were terpenoids (mono- and sesquiterpenoids), while in quince fruit, esters were most prevalent. We hypothesise that (specific) esters could play a major role why WHF adults chose quince more than (or at least the same as) persian walnut, since tephritids have a highly ester tuned olfactory circuitry^16,39,40^. Freshly emerged female adults of tephritid flies have undeveloped ovaries, and rely on additional feeding to gain maturity^41–44^. It should be a straightforward connection, that tephritid adults feed on the fruit itself, however, this relationship could have additional layers to it. For example the general attraction between host plants and the fruit fly *Drosophila melanogaster* is greatly mediated by the epiphytic yeasts inhabiting the fruit surface^45^. As a matter of fact, the formation of “fruity” and “flowery” smell is somewhat attributed to these microorganisms^46,47^, some of the key components of fruit-flower smell are esters and their derivatives^48^. The importance of epiphytic yeasts in the context of insect-yeast preference was investigated also in tephritid flies. The olive fruit fly (*Bactocera oleae*) showed different attraction towards various yeast species, and yeast baited traps caught adults on on-field experiments^49^, indicating the important role that yeasts have. The interaction between fruit surface dwelling yeasts and fruit flies is mutualistic, as yeast species provide nutrients for fruit flies, while fliesspread these microorganisms to new fruit hosts^50^. In our case, virgin WHF adults showed great interest in quince fruits. The fruit surface of pomaceous plants provide a habitat for a variety of yeast species^51^ which could provide nutrients for tephritids^49,52^. However, the surface of *Juglans regia* fruits—which is considered economically the most relevant host plant of WHF—seem to lack yeast species^53,54^, that could provide nutritious rich food source for freshly emerged WHF adults. The husk of *Juglans* sp. contain antibacterial and antifungal compounds^55^ that may affect the epidermal microbiome. However it cannot be stated that the surface of walnuts are absent of micro fungi; Pardatscher and Schweigkofler^53^ did extensive microbe profiling of persian walnuts in Italian regions and concluded that from the 3880 microbial cultures, 96,4% were isolated as fungi,categorised into 30 genera. From the ~ 3900 isolates, only 14 were categorised as “yeasts”.

The question arises then, “What do WHF adults feed on?”. Tephritid flies feed on a large variety of food sources^4^.Honeydew could be an important source of food for tephritids, as it contains carbohydrates, vitamins and amino acids^56^, while bird droppings could supply a valuable source of nitrogen for fruit flies^26^. The work on an artificial diet has shown that adults require a protein source for improved survival and egg production^4^. In the case of *R. pomonella* the addition of unhydrolysed yeast prolonged survival and fecundity^57^. The work of Hagen and Finney^58^ also declared that the protein hydrolysate in the diet of *B. dorsalis*, *B. cucurbiate* and *C. capitata* greatly increased fecundity. In this sense, it is plausible that the protein need of *R. completa* could be provided from the protein content of epiphytic yeast on fruits while the insect is foraging for food. Other microorganisms such as bacteria could serve as an important food source for WHFs. There are fruit fly species where bacteria based food supplements significantly enhanced the ovarian development^42^. Later Drew and Lloyd^59^ also reported that bacteria introduced on peaches by *Bactocera tryoni* and *B. neohumeralis* was an important food source for adult flies. Furthermore the odour of many bacterial strains seem to be attractive for fruit flies, i.e. *Enterobacter agglomerans*, which is very commonly isolated from bird dropping^60^. Tsiropoulos^61^ eliminated the microflora with antibiotics from the surface of walnut fruits, and found that the survival, pre-oviposition period, fecundity and fertility of adults was adverse on sterilised diet. With this in mind it could be possible that epiphytic bacteria of *Juglans* sp. could be beneficial for *R. completa* female sexual maturation.

The specialisation to walnut appears to be highly pronounced from an evolutionary perspective. Most tephritid fly species prefer host plants for feeding and oviposition that have large, fleshy, high sugar content fruits. Majority of the North American Rhagoletis species belong to the *pomonella*, *tabellaria*, *ribicola*, *cingulata*, and *suavis* gropus [walnut infesting species belong to *suavis* group]^62^. In the publication of Smith and Bush^62^ there is given an extensive host plant range of each taxonomic group. To give a brief summary, the *pomonella* group’s host consist of *Crataegus mollis*, *Malus pumila*, *Vaccinium corymbosum*, V. *angustifolium*, *Cornus florida*, *C. obliqua*; *tabellaria* group: *Cornus stolonifera*, *C. foemina*, *Vaccinium parvifolium*, *Disporum trachycarpum*, *Sheperdia argentea*; *ribicola*: *Ribes* sp. *Mahonia* sp. (*repens*?); *cingulata*: *Prunus serotina*, *P. cerasus*, *Osmanthus americanus*, *Chionanthus virginicus*; and *suavis* has *Juglans nigra*, *J. regia*, *Juglans* sp. as hosts. It is very important to compare these fruits and see the similarities. All of them are “round, berry-like”, with softening tissues as fruit senescence progresses. The colour of these fruits range from blue, different hues of orange, red, blackish red. From these characteristics, the fruits of various walnuts seem to be an extreme outlier, with little to no similarities with the other groups’ host-fruits. Since all of the North American Rhagoletis species are placed on the same dendrographic branch, meaning these species are in the closest relationship with each other. Our hypothesis based on the properties of these fruits is that the ancient host plant of North American Rhagoletis must have had a closer feature towards berry-like or pomaceous fruits. We think that *R. completa* did in fact exploit other fruits, and later specialised on walnuts. This is reinforced by our electrophysiological findings that *R. completa* gave signals during EAD measurements to volatiles that were not present in the headspace of walnut fruit or foliage samples. The same was true, when the signals of the maxillary palpi were examined. There were numerous volatiles that were absent in walnut fruit, yet induced well defined electrophysiological signals. Some of these unique quince volatiles could either be sensed both by the antennae and by palpi, or by these organs separately. The majority of these volatiles were esters, to which the olfactory neural complex of antennae and palpi of tephritids are highly tuned to^16,40^.These findings mean that *R. completa* has a wide range of olfactory receptors that can also activate to non-walnut volatiles, which could mean that at some point in evolution the host plant range of *R. completa* was different. The other possibility is that to this day WHF utilises its full receptor array to locate other plants (or microorganisms) than walnut when foraging. This is indicated from the behavioural experiments, that quince fruit was in fact significantly more appealing to virgin females than walnut fruits, while male specimens did not discriminate between them. It is important to note, that specimens could not visually see the two fruit samples, which means the choice was mediated only through chemical channels.

There was some difference in the perception capability between sexes in EAD recordings. While there was no significant contrast when walnut samples were tested, male individuals tended to give more antennal signals to volatiles as compared to females when a quince sample was analysed. Fruit flies mark the surface of their host fruit with a special chemical blend to signalise and to reduce the likelihood of oviposition on the same fruit by other conspecifics^63^. This is done usually by females in the Rhagoletis genus, however there are exeptions. The host marking behaviour of females in the *suavis* group is inconsistent and is hypothesised that males are the one who are marking the hosts. It is still not fully understood if every *suavis* species’s male mark, but *R. boycei* males frequently touch the host fruit with their proctigers, leaving a clear viscous substance on the fruit in the process. It is very important to note, that females of *R. boycei* preferred to attempt oviposition in the vicinity of male marks, as opposed to areas where no male mark was left^64^, suggesting that male-marks have an incentive effect on oviposition. It is still not known if *R. completa* males mark their host plant or not, but it may be the case with quince, that males incorrectly mark a fruit which may additionally deter females from their natural host. It is known in Rhagoletis, that resource distribution can have an influence on mating sites. If resources are abundant, then males can form leks away from oviposition sites where they call for females in order to reduce the intraspecific mating competition^65^. It could be that males were responsible for deterring females from walnut to quince, by calling and possible host-marking on quince, which ultimately influenced females to wrongly oviposit on a dead-end host. The physiology of females can also affect behaviour in various ways. Females with high egg load will search for host plants more quickly, discriminate less in host-plant hierarchy, deposit larger clusters of eggs, etc.^66^. In the context of walnut exploiting species, *R. juglandis* females with high egg load hyper parasitised walnut fruits that were already utilised by conspecifics, disregarding their host marking pheromone cue^67^. We did not find literature if egg load can alter *R. completa* female behaviour, yet it could have been an influencing factor in this case. However there is no mention in literature that high female egg load promoted novel host plant seeking, or the choice of lethal host for offsprings in Rhagoletis species.

There were also dissimilarities between male and female palpus recordings. The palpi of females gave response to a slightly wider range of volatiles compared to males. This difference could arrive from the fact that females need specific nutritional needs and responses to food sources can vary, with females often showing a stronger reliance on certain types of food for reproductive success compared to males^68^. This could mean that females need to discriminate more precisely from volatile cues in their environment in order to find the most suitable feeding source, thus having the need to perceive a wider range of chemical cues through their palpi. Furthermore, a part of the host acceptance process by females is the tactile observation of fruit, where surface chemicals are sampled by the receptors found on the tarsi and palpi of the insect^35^. It is not implausible that the palpi of female WHF are active to more volatiles compared to males, since they use these organs in host plant verification before oviposition. Herrera et al.^40^ recently did an extensive comparison of tephritid antennal and palpal response to different fruit and food volatiles and concluded that overall the antennae is more sensitive towards fruit volatiles, while palpal receptors are more tuned to food related odors. Similarly, we also found that from the volatilome of quince fruit, 32 volatiles were detected by the antennae and 17 by the maxillary palpi, with 10 overlapping compounds (all of them esters). From the total walnut fruit compounds, the antennae gave response to 18 compounds, while palpi actively sensed 12, with 7 overlapping. The number of detected volatiles between antennae and palpi seems to be more even in the case of walnut. Herrera et al. suggested that tephritids with similar ecology had a similar palpal response pattern towards food volatiles while phylogenetic relation is a less decisive factor. This, in the context of *R. completa* could be quite intriguing. If the palpal responses of WHF are similar to those of other Rhagoletis species, it might suggest that the dietary needs of WHF could also be similar to those of other tephritid flies in the same genus and thus search for similar plants for food-source. However no such data exists that shows the palpi active compounds of the Rhagoletis genus. Based on the data presented by Herrera et al. we found matching compounds that elicited palpal response from *R. completa* and the species they examined (*B. dorsalis*, *C. capitata* and *Z. cucuberitae*). Although the overall overlapping volatiles between the two experiments are few, certain volatiles appear to be conserved as palpal-active in tephritid species, but as suggested by Herrera et al., ecology is more important than phylogenetic relation. It is important to note that, despite the detection of these compounds by various tephritid species’ palpal and antennal olfactory receptors, the resulting neurological processing could lead to species-specific behavioral outcomes.

In conclusion the fact that a highly specialised fruit fly species such as *Rhagoletis completa* in its natural habitat chose an ecologically outlier fruit like quince as ovipositional host is quite intriguing. What makes this “mistake” more than just a coincidence, is that this event was observed also in the following year. Furthermore the behavioural display of WHFs was identical to what occurs on their natural host-plant during mating and ovipositing. The electrophysiological screenings proved that both sexes of WHF adults can antenally perceive unique non-host volatiles, and in behavioural experiments, quince was more attractive for unmated females, than walnut fruits. A concrete explanation to this phenomenon cannot be given at this point, but we have hypothesised certain factors that could have each contributed to this outcome. our electrophysiological readings of antennae and maxillary palp suggests, that the olfactory receptors are not only tuned for walnut volatiles.Furthermore, in Y-tube choice preference assays virgin females favoured quince compared to walnut fruits, which means this choice was mediated by certain volatiles originating from quince. To unveil this mystery better, one of the most important future plans is to investigate if the fruit preference for females shifts towards walnut fruits if mating has occurred. Likewise, further olfactory/neural examinations are required on the quince-associated WHF adults, to conclude if these specimens differ in any aspect from the original, walnut associated ones.

## Supplementary Information

Supplementary Information.

## Data Availability

Raw data of experiments and recordings have been uploaded and publicly available at figshare (https://figshare.com/s/6e77a15c5bf53ecd2cde).

## Declarations

### Research involving plant collection

The plant species *Juglans regia* and *Cydonia oblonga* used in the present study are commonly found and are not protected in Hungary. Therefore, they can be freely collected and used without permit or approval from national authorities under Hungarian law 13/2001. (V. 9.).
