# Supplementary material for: Oviposition behaviour of Rhagoletis completa on dead end host Cydonia oblonga
Source: Sci Rep. 2025 Apr 1;15:11070. doi: 10.1038/s41598-025-88677-y (PMC11961671; doi:10.1038/s41598-025-88677-y)
Supplement: Supplementary file 1 — Supplementary Information. [file 41598_2025_88677_MOESM1_ESM.pdf]

**Supplementary Information for the manuscript titled:**

# Oviposition behaviour of Walnut husk fly (*Rhagoletis completa*) on dead-end host, European quince (*Cydonia oblonga)*

**Authors and affiliations:**

SÁNDOR KECSKEMÉTI[1], ANNA LAURA ERDEI[1,2], JANKA SIMON[3], BALÁZS KISS[3], BÉLA PÉTER MOLNÁR[1]

[1] *Department of Chemical Ecology, Plant Protection Institute, Centre for Agricultural Research, HUN-REN, Budapest, Hungary*
[2]*Department of Plant Protection Biology, Swedish University of Agricultural Sciences, Alnarp, Sweden*
[3]*Department of Zoology, Plant Protection Institute, Centre for Agricultural Research, HUN-REN, Budapest, Hungary*

**Corresponding author: kecskemeti.sandor@atk.hun-ren.hu*

ORCID information:

Anna Laura Erdei: 0000-0002-7819-1287

Sándor Kecskeméti: 0000-0002-3952-8174

Balázs Kiss: 0000-0003-2511-9094

Béla Péter Molnár: 0000-0002-3192-0868

Janka Simon: 0000-0001-9601-0227

Table S1.) Total volatile profile analyses of European quince (*Cydonia oblonga*) and English walnut (*Juglans regia*) fruits and foliage using GC-MS. Detected compounds are in ascending order based on calculated Kovats retention indices ($RI_a$) and NIST library records ($RI_b$) and numbered (#) for quick reference in the manuscript (i.e.: #39 references amyl acetate in a figure). Compounds marked with asterisk were tested with synthetic compounds (see Table S2).

| | | | | | | Relative amount (%) | | | |
|---|---|---|---|---|---|---|---|---|---|
| | | | | | | ***Cydonia oblonga*** | | ***Juglans regia*** | |
| **#** | **$RI_a$** | **$RI_b$** | ***Compounds** | **CAS** | **Literature reference** | ***Fruit*** | ***Foliage*** | ***Fruit*** | ***Foliage*** |
| **1** | 758 | 749 | tiglic aldehyde | 497-03-0 | Q2 | 0,05 ± 0,01 | | | |
| **2** | 763 | 759 | 2,3,4-trimethyl-pentane | 565-75-3 | | | 0,03 ± <0,001 | | |
| **3** | 767 | 756 | *ethyl isobutyrate | 97-62-1 | Q1 | 1,54 ± 0,3 | 0,03 ± 0,01 | | |
| **4** | 777 | 770 | toluene | 108-88-3 | | 0,31 ± 0,02 | 0,75 ± 0,16 | 0,11 ± 0,01 | 0,03 ± <0,001 |
| **5** | 778 | 770 | *isobutyl acetate | 110-19-0 | Q2,Q3 | 2,66 ± 0,38 | | | |
| **6** | 782 | 777 | methyl-2-methyl butyrate | 868-57-5 | | 0,03 ± <0,001 | | | |
| **7** | 787 | 785 | diethyl carbonate | 105-58-8 | | 0,3 ± 0,08 | | | |
| **8** | 789 | 786 | 3-hexanone | 105-58-8 | | | 0,06 ± | 0,01 ± <0,001 | 0,01 ± <0,001 |
| **9** | 791 | 790 | 2-hexanone | 591-78-6 | | 0,06 ± 0,01 | 0,01 ± <0,001 | 0,08 ± 0,01 | 0,02 ± <0,001 |
| **10** | 796 | | Unknown compound 1 (ester) | | | | | 0,01 ± <0,001 | |
| **11** | 799 | 802 | *3-hexanal | 4440-65-7 | | | 3,29 ± 0,89 | | |
| **12** | 801 | 801 | *ethyl butyrate | 105-54-4 | Q2 | 2,92 ± 0,67 | | | |
| **13** | 801 | 801 | hexanal | 66-25-1 | W1 | | 0,29 ± 0,05 | 0,1 ± 0,02 | 0,04 ± 0,01 |
| **14** | 813 | 809 | *butyl acetate | 123-86-4 | Q2 | 1,28 ± 0,18 | | 0,01 ± <0,001 | 0,001 ± <0,001 |
| **15** | 821 | 820 | 2,4-dimethylheptane | 2213-23-2 | | | 0,23 ± 0,04 | | |
| **16** | 821 | | Unknown compound 2 | | | | | 0,02 ± <0,001 | 0,02 ± <0,001 |
| **17** | 830 | | Unknown compound 3 (acetate) | | | 0,01 ± <0,001 | | | |
| **18** | 836 | 857 | 1,2,4,4-tetramethylcyclopentene | 65378-76-9 | | | | 0,02 ± <0,001 | 0,03 ± <0,001 |
| **19** | 842 | 835 | ethyl (*Z*)-2-butenoate | 6776-19-8 | Q2 | 0,99 ± 0,17 | | | |
| **20** | 844 | | Unknown compound 4 (ester) | | | | 0,02 ± <0,001 | | |
| **21** | 848 | 850 | ethyl 2-methylbutyrate | 7452-79-1 | Q2,Q3 | 6,72 ± 0,83 | | 0,01 ± <0,001 | 0,05 ± <0,001 |
| **22** | 850 | 855 | *(*E*)-3-hexen-1-ol | 928-97-2 | | | 0,08 ± 0,01 | | |
| **23** | 850 | 857 | *ethyl isovalerate | 108-64-5 | | 2,05 ± 0,29 | | 0,01 ± <0,001 | 0,17 ± 0,05 |
| **24** | 853 | 855 | *(*Z*)-3-hexen-1-ol | 928-96-1 | Q1,Q3,W1,W2 | | 12,04 ± 1,84 | 0,01 ± <0,001 | 0,16 ± 0,04 |
| **25** | 863 | 868 | ethylbenzene | 100-41-4 | | 0,04 ± 0,01 | 0,73 ± 0,17 | 0,03 ± <0,001 | 0,02 ± <0,001 |
| **26** | 865 | 867 | 1-hexanol | 111-27-3 | Q1,Q2 | | 0,25 ± 0,04 | | |
| **27** | 866 | 870 | 1-methoxy-2-propyl acetate | 108-65-6 | | | | 0,03 ± <0,001 | |
| **28** | 870 | 865 | *p*-xylene | 106-42-3 | | 0,14 ± 0,03 | 2 ± 0,08 | 0,07 ± 0,01 | 0,03 ± <0,001 |
| **29** | 875 | 876 | *isoamyl acetate | 123-92-2 | Q2 | 3,34 ± 0,5 | | | |
| **30** | 878 | 882 | 2-methylbutyl acetate | 624-41-9 | Q3 | 4,01 ± 0,55 | | | |
| **31** | 884 | | 2-methyl-1-nonene-3-yne | 70058-00-3 | | | | 0,13 ± 0,01 | 0,2 ± 0,01 |
| **32** | 889 | 890 | 2-heptanone | 110-43-0 | Q3 | 0,01 ± <0,001 | | 0,01 ± <0,001 | 0,001 ± <0,001 |

| | | | | | | | | | |
|---|---|---|---|---|---|---|---|---|---|
| **33** | 893 | 893 | styrene | 100-42-5 | | | 0,17 ± 0,01 | 0,03 ± <0,001 | 0,01 ± <0,001 |
| **34** | 896 | 896 | *o*-xylene | 95-47-6 | | 0,05 ± 0,01 | 0,32 ± 0,05 | 0,03 ± <0,001 | 0,01 ± <0,001 |
| **35** | 900 | 898 | *ethyl pentanoate | 539-82-2 | | 0,2 ± 0,04 | | | |
| **36** | 900 | 905 | nonane | 111-84-2 | | | | | 0,02 ± <0,001 |
| **37** | 901 | 902 | heptanal | 111-71-7 | | | | 0,07 ± 0,01 | |
| **38** | 908 | 912 | 2,7,7-trimethyl-bicyclo[2.2.1]hept-2-ene | 514-14-7 | | | | 0,13 ± 0,01 | 0,14 ± 0,02 |
| **39** | 912 | 911 | *amyl acetate | 628-63-7 | Q2,Q3,W2 | 0,09 ± 0,01 | | | |
| **40** | 921 | 921 | *2-methylbut-2-en-1-yl acetate | 33425-30-8 | Q2 | 1,38 ± 0,24 | | | |
| **41** | 923 | 921 | 5,5-dimethyl-1-vinylbicyclo[2.1.1]hexane | 16626-39-4 | | | | | 0,02 ± <0,001 |
| **42** | 926 | 924 | tricyclene | 508-32-7 | | | | 0,3 ± 0,04 | 0,31 ± 0,05 |
| **43** | 930 | 929 | 3-thujene | 2867.02.05 | W1,W3 | | | 0,33 ± 0,05 | 0,4 ± 0,03 |
| **44** | 933 | 945 | ethyl 3-hydroxybutyrate | 5405-41-4 | | 0,02 ± 0,01 | | | |
| **45** | 937 | 939 | *$\alpha$-pinene | 80-56-8 | Q2,W1,W3 | | 0,93 ± 0,1 | 22,32 ± 2,76 | 18,51 ± 2,81 |
| **46** | 940 | 938 | *ethyl tiglate | 5837-78-5 | Q2,Q3 | 3,94 ± 1,11 | | | |
| **47** | 944 | | Unknown compound 5 | | | | | 0,12 ± 0,02 | |
| **48** | 945 | 944 | isobutyl 2-methylbutanoate | 2445-67-2 | | 0,05 ± 0,01 | | | |
| **49** | 946 | | Unknown compound 6 | | | | | | 2,9 ± 0,49 |
| **50** | 948 | 951 | propyl 3-methylbutyrate | 557-00-6 | | 0,02 ± <0,001 | | | 0,07 ± 0,01 |
| **51** | 954 | 952 | camphene | 79-92-5 | W3 | | | 4,35 ± 0,99 | 3,48 ± 0,56 |
| **52** | 958 | 955 | 4(10)-thujadiene | 36262-09-06 | | | | 0,22 ± 0,02 | 0,32 ± 0,05 |
| **53** | 964 | 958 | 1-ethyl-3-methylbenzene | 620-14-4 | | 0,05 ± 0,01 | 0,18 ± 0,02 | | |
| **54** | 964 | | Unknown compound 7 | | | | | 0,01 ± <0,001 | |
| **55** | 965 | | Unknown compound 8 | | | | | | 0,001 ± <0,001 |
| **56** | 969 | 973 | *sabinene | 3387-41-5 | W1,W2,W3 | | 0,03 ± <0,001 | 0,04 ± 0,01 | 0,01 ± <0,001 |
| **57** | 981 | 978 | *$\beta$-pinene | 127-91-3 | W1,W2,W3 | | 0,3 ± 0,04 | 29,66 ± 4,85 | 22,61 ± 2,04 |
| **58** | 986 | 985 | ethyl-5-hexanoate | 500027-11-2 | Q3 | 0,11 ± 0,01 | | | |
| **59** | 992 | 992 | *$\beta$-myrcene | 123-35-3 | W1,W2,W3 | 0,1 ± 0,01 | 0,24 ± 0,03 | 2,54 ± 0,58 | 2,9 ± 0,49 |
| **60** | 996 | 998 | *ethyl hexanoate | 123-66-0 | Q1,Q2,Q3 | 12,08 ± 1,46 | | 0,13 ± 0,02 | 0,07 ± 0,01 |
| **61** | 997 | 997 | 1,2,3-trimethyl-benzene | 526-73-8 | | | 0,25 ± 0,08 | | |
| **62** | 1003 | 1004 | octanal | 124-13-0 | Q1,Q2,W2,W3 | | | 0,1 ± 0,02 | |
| **63** | 999 | 1000 | decane | 124-18-5 | | | 0,28 ± 0,02 | | |
| **64** | 1006 | 1005 | *(*Z*)-3-hexenyl-acetate | 3681-71-8 | Q1,Q2,W1,W2,W3 | 0,28 ± 0,03 | 62,57 ± 12,77 | 0,71 ± 0,11 | 3,48 ± 0,56 |
| **65** | 1008 | 1005 | $\alpha$-phellandrene | 9-83-2 | W3 | | | 0,77 ± 0,14 | |
| **66** | 1012 | 1011 | *hexyl acetate | 142-92-7 | Q2,Q3,W2 | 1,34 ± 0,15 | 0,66 ± 0,11 | | |
| **67** | 1013 | | Unknown compound 9 (ester) | | | | | | 0,02 ± <0,001 |
| **68** | 1014 | 1012 | 3-carene | 13466-78-9 | | | 0,56 ± 0,02 | | |
| **69** | 1015 | | Unknown compound 10 | | | | | | 0,02 ± <0,001 |
| **70** | 1017 | | Unknown compound 11 | | | | | | 0,02 ± <0,001 |
| **71** | 1020 | 1017 | $\alpha$-terpinene | 99-86-5 | W2,W3 | | 0,1 ± 0,02 | 0,77 ± 0,13 | 0,8 ± 0,04 |
| **72** | 1022 | | Unknown compound 12 (alkane) | | | | 0,14 ± 0,02 | | |
| **73** | 1023 | | Unknown compound 13 (ester) | | | 0,001 ± <0,001 | | | |
| **74** | 1027 | 1026 | *p*-cymene | 99-87-6 | W1,W2,W3 | 0,04 ± 0,01 | 0,51 ± 0,07 | 1,03 ± 0,19 | 1,14 ± 0,14 |
| **75** | 1032 | 1035 | *limonene | 138-86-3 | Q2,Q3,W1,W2,W3 | 0,07 ± 0,01 | 1,47 ± 0,07 | 10,16 ± 1,83 | 10,47 ± 2,84 |
| **76** | 1035 | 1035 | propyl tiglate | 61692-83-9 | Q2 | 0,02 ± <0,001 | | | |

| | | | | | | | | | |
|---|---|---|---|---|---|---|---|---|---|
| **77** | 1037 | 1046 | *eucalyptol | 470-82-6 | | | | 1,29 ± 0,11 | 1,33 ± 0,13 |
| **78** | 1038 | | Unknown compound 14 | | | | | 0,1 ± 0,3 | |
| **79** | 1041 | 1043 | 2-heptyl acetate | 0112-06-01 | | 0,01 ± <0,001 | | | |
| **80** | 1043 | 1043 | ethyl 2-hexenoate | 1552-67-6 | | 0,15 ± 0,03 | | | |
| **81** | 1043 | | Unknown compound 15 | | | | | | 0,01 ± <0,001 |
| **82** | 1050 | 1040 | *(Z)-β-ocimene | 3338-55-4 | Q3,W1,W2 | | 0,5 ± 0,15 | 3,98 ± 0,04 | 3,94 ± 0,55 |
| **83** | 1056 | | Unknown compound 16 | | | | | | 0,01 ± <0,001 |
| **84** | 1058 | | Unknown compound 17 (alkane) | | | 0,01 ± <0,001 | 0,17 ± 0,05 | | |
| **85** | 1059 | | Unknown compound 18 | | | | | | 0,001 ± <0,001 |
| **86** | 1062 | | Unknown compound 19 | | | | 0,12 ± 0,02 | | |
| **87** | 1062 | 1060 | *γ-terpinene | 99-85-4 | W1,W2,W3 | | | 0,83 ± 0,03 | 0,81 ± 0,2 |
| **88** | 1064 | | Unknown compound 20 | | | | 0,15 ± 0,03 | | |
| **89** | 1066 | | Unknown compound 21 (ester) | | | 0,001 ± <0,001 | | | |
| **90** | 1070 | 1097 | (Z)-pent-2-enyl butyrate | 42125-13-3 | | 0,02 ± <0,001 | | | |
| **91** | 1071 | 1072 | (E)-4-thujanol | 546-79-2 | | | 0,03 ± <0,001 | 0,08 ± 0,02 | 0,03 ± 0,01 |
| **92** | 1076 | 1075 | (Z)-linalool oxide | 5989-33-3 | | 0,001 ± <0,001 | | | |
| **93** | 1079 | | Unknown compound 22 | | | | | | 0,01 ± <0,001 |
| **94** | 1080 | | Unknown compound 23 | | | | 0,04 ± 0,01 | | |
| **95** | 1083 | | Unknown compound 24 | | | | | | 0,01 ± <0,001 |
| **96** | 1088 | | ethyl-6 heptanoate | 106-30-9 | | 0,03 ± <0,001 | | | |
| **97** | 1088 | 1095 | (Z)-verbenol | 1845-30-3 | W1 | | | 0,06 ± 0,01 | 0,03 ± <0,001 |
| **98** | 1092 | 1093 | terpinolene | 586-62-9 | W1,W2,W3 | | 0,07 ± 0,01 | 1,17 ± 0,22 | 1,11 ± 0,05 |
| **99** | 1094 | 1094 | propyl hexanoate | 626-77-7 | Q3 | 0,06 ± 0,01 | | | |
| **100** | 1097 | 1097 | *ethyl heptanoate | 106-30-9 | Q2,Q3 | 2,21 ± 0,45 | | | |
| **101** | 1098 | 1093 | rosefuran | 15186-51-3 | | | | | 0,03 ± <0,001 |
| **102** | 1099 | 1101 | 3-methylbut-2-enyl butanoate | 89026-29-9 | | 0,52 ± 0,11 | | | |
| **103** | 1100 | 1104 | *linalool | 78-70-6 | W1,W2,W3 | | | 0,2 ± 0,02 | 0,07 ± 0,01 |
| **104** | 1104 | 1102 | nonanal | 124-19-6 | Q1,Q3,W1 | 0,03 ± 0,01 | 0,17 ± 0,03 | 0,39 ± 0,09 | |
| **105** | 1105 | 1103 | isoamyl isovalerate | 659-70-1 | | | | | 0,07 ± 0,02 |
| **106** | 1108 | 1107 | 2,6,6-trimethylbicyclo[3.2.0]hept-2-en-7-one | | | | 0,13 ± 0,01 | | 0,03 ± <0,001 |
| **107** | 1111 | 1110 | *heptyl acetate | 112-06-0 | Q3 | 0,07 ± 0,02 | | | |
| **108** | 1117 | 1117 | *(E)-4,8-dimethyl-1,3,7-nonatriene | 19945-61-0 | W1,W2,W3 | | 4,93 ± 0,35 | 0,23 ± 0,07 | 0,13 ± 0,04 |
| **109** | 1122 | 1113 | *thujone | 76231-76-0 | | | | 0,09 ± 0,01 | 0,16 ± 0,02 |
| **110** | 1124 | 1126 | methyl octanoate | 0111-11-5 | Q2,Q3 | 0,08 ± 0,01 | | | |
| **111** | 1131 | 1129 | chrysanthenone | 0473-06-03 | W3 | | | 0,59 ± 0,04 | 0,37 ± 0,06 |
| **112** | 1139 | | Unknown compound 25 | | | | | 0,03 ± <0,001 | |
| **113** | 1146 | 1140 | pinocarveol | 5947-36-4 | W2,W3 | | | 1,17 ± 0,12 | 0,8 ± 0,21 |
| **114** | 1150 | 1146 | (E)-verbenol | 1820-09-03 | | | | 0,7 ± 0,11 | 0,47 ± 0,1 |
| **115** | 1160 | | Unknown compound 26 (ester) | | | 0,01 ± <0,001 | | | |
| **116** | 1164 | 1156 | sabinaketone | 513-20-2 | W2,W3 | | | | 0,01 ± <0,001 |
| **117** | 1168 | 1167 | benzyl acetate | 0140-11-4 | Q2,Q3 | 0,01 ± <0,001 | | | |
| **118** | 1171 | 1165 | pinocarvone | 30460-92-5 | W1,W2,W3 | | | 0,9 ± 0,24 | 0,27 ± 0,05 |
| **119** | 1172 | 1176 | 1-nonanol | 0143-08-08 | Q2 | 0,03 ± <0,001 | | | |
| **120** | 1174 | 1167 | endo-borneol | 507-70-0 | W3 | | | | 0,04 ± 0,01 |

| | | | | | | | | | |
|---|---|---|---|---|---|---|---|---|---|
| **121** | 1175 | 1176 | ethyl benzoate | 93-89-0 | | 0,02 ± <0,001 | | | |
| **122** | 1181 | 1143 | ethyl 3-(methylthio)-(*E*)-2-propenoate | 136115-65-6 | | 0,08 ± 0,01 | | | |
| **123** | 1183 | 1182 | 3-pinanone | 15358-88-0 | | | | 2,76 ± 0,51 | 2,77 ± 0,65 |
| **124** | 1187 | 1197 | 2,6-dimethyl-3,7-octadiene-2,6-diol | 13741-21-4 | | | 0,13 ± 0,02 | | |
| **125** | 1189 | 1187 | ethyl 7-octenoate | 35194-38-8 | | 0,97 ± 0,2 | | | |
| **126** | 1192 | | Unknown compound 27 | | | | | 0,19 ± 0,02 | 0,02 ± <0,001 |
| **127** | 1200 | 1200 | dodecane | 112-40-3 | | | 0,44 ± 0,08 | | |
| **128** | 1201 | 1195 | *ethyl octanoate | 106-32-1 | Q1,Q3 | 18,03 ± 2,35 | | | |
| **129** | 1203 | 1193 | myrtenol | 19894-97-4 | W1,W2,W3 | | | 1,47 ± 0,19 | 0,42 ± 0,13 |
| **130** | 1207 | 1206 | decanal | 112-31-2 | Q2,W3 | 0,02 ± <0,001 | | 0,3 ± 0,03 | |
| **131** | 1211 | | Unknown compound 28 (ester) | | | 0,08 ± 0,03 | | | |
| **132** | 1215 | | Unknown compound 29 | | | | | 0,58 ± 0,04 | 0,36 ± 0,07 |
| **133** | 1224 | 1229 | (*E*)-carveol | 2102-58-1 | W2,W3 | | | 0,1 ± 0,01 | 0,05 ± <0,001 |
| **134** | 1232 | 1219 | isopropyl octanoate | 5458-59-3 | | 0,01 ± <0,001 | | | |
| **135** | 1233 | 1223 | (*Z*)-3-hexenyl-α-methylbutyrate | 53398-85-9 | | | | | 0,16 ± 0,02 |
| **136** | 1237 | 1238 | (*Z*)-3-hexenyl isovalerate | 35154-45-1 | | 0,001 ± <0,001 | | | |
| **137** | 1247 | 1246 | ethyl-(*E*)-2-octenoate | 7367-82-0 | Q2 | 0,47 ± 0,08 | | | |
| **138** | 1251 | 1254 | carvone | 99-49-0 | W2,W3 | | | 0,16 ± 0,03 | 0,16 ± 0,02 |
| **139** | 1258 | 1258 | phenylethyl acetate | 103-45-7 | | 0,04 ± 0,01 | | 0,05 ± 0,01 | 0,04 ± <0,001 |
| **140** | 1264 | | Unknown compound 30 | | | | | 0,02 ± <0,001 | |
| **141** | 1269 | | Unknown compound 31 | | | | | 0,03 ± <0,001 | 0,001 ± <0,001 |
| **142** | 1272 | 1273 | 1-decanol | 112-30-1 | | 0,01 ± <0,001 | | | |
| **143** | 1273 | | Unknown compound 32 | | | | | 0,04 ± <0,001 | |
| **144** | 1276 | | Unknown compound 33 (ester) | | | 0,03 ± 0,01 | | | |
| **145** | 1280 | | Unknown compound 34 | | | | | 0,16 ± 0,02 | 0,05 ± <0,001 |
| **146** | 1281 | | Unknown compound 35 (alkane) | | | | 0,19 ± 0,03 | | |
| **147** | 1288 | | Unknown compound 36 (ester) | | | 0,01 ± 0,01 | | | |
| **148** | 1292 | 1290 | propyl octanoate | 624-13-5 | Q2 | 0,09 ± 0,01 | | | |
| **149** | 1292 | 1288 | bornyl acetate | 76-49-3 | W2,W3 | | | 0,29 ± 0,09 | 0,11 ± 0,02 |
| **150** | 1295 | 1294 | *ethyl nonanoate | 123-29-5 | | 0,79 ± 0,11 | | | |
| **151** | 1299 | 1300 | tridecane | 629-50-5 | W3 | | 0,12 ± 0,02 | | 0,01 ± <0,001 |
| **152** | 1300 | | Unknown compound 37 | | | | | 0,02 ± <0,001 | |
| **153** | 1305 | | Unknown compound 38 | | | | | 0,17 ± 0,02 | |
| **154** | 1306 | | Unknown compound 39 | | | | | | 0,01 ± <0,001 |
| **155** | 1308 | | Unknown compound 40 | | | | | 0,03 ± 0,01 | |
| **156** | 1315 | | Unknown compound 41 | | | | | 0,17 ± 0,02 | |
| **157** | 1319 | | Unknown compound 42 | | | | | 0,05 ± 0,01 | |
| **158** | 1320 | | Unknown compound 43 | | | | | | 0,001 ± <0,001 |
| **159** | 1332 | 1331 | ethyl-3-hydroxyoctanoate | 7367-90-0 | | 0,1 ± 0,01 | | | |
| **160** | 1333 | | Unknown compound 44 | | | | | | 0,07 ± 0,01 |
| **161** | 1337 | | Unknown compound 45 | | | | | | 0,001 ± <0,001 |
| **162** | 1348 | 1348 | isobutyl octanoate | 5461.06.03 | Q3 | 0,03 ± 0,01 | | | |
| **163** | 1348 | 1354 | γ-terpinyl acetate | 10235-63-9 | | | | 0,11 ± <0,001 | 0,001 ± <0,001 |
| **164** | 1354 | | Unknown compound 46 | | | | | | 0,02 ± 0,01 |

| | | | | | | | | | |
|---|---|---|---|---|---|---|---|---|---|
| **165** | 1359 | 1354 | *α*-cubebene | 17699-14-8 | W2,W3 | | | 0,01 ± <0,001 | 0,05 ± 0,01 |
| **166** | 1365 | 1365 | neryl acetate | 0141-12-8 | W2,W3 | | | 0,04 ± <0,001 | 0,04 ± 0,01 |
| **167** | 1373 | | Unknown compound 47 (ester) | | | 0,02 ± <0,001 | | | |
| **168** | 1375 | | Unknown compound 48 | | | | | | 0,01 ± <0,001 |
| **169** | 1380 | | Unknown compound 49 | | | | | | 0,03 ± 0,01 |
| **170** | 1381 | 1382 | ethyl (*E*)-4-decenoate | 76649-16-6 | Q1,Q2 | 2,01 ± 0,39 | | | |
| **171** | 1384 | | Unknown compound 50 | | | | | 0,06 ± 0,01 | 0,03 ± <0,001 |
| **172** | 1388 | 1378 | copane | 3856-25-5 | Q2 | | | 0,01 ± <0,001 | 0,08 ± 0,01 |
| **173** | 1389 | 1392 | *ethyl decanoate | 110-38-3 | Q1,Q2,Q3 | 0,45 ± 0,06 | | | |
| **174** | 1391 | 1391 | 1-tetradecene | 1120-36-1 | | | | 0,01 ± <0,001 | 0,07 ± 0,01 |
| **175** | 1397 | 1386 | *β*-bourbonene | 5208-59-3 | W1,W2,W3 | 7,53 ± 1,02 | | 0,24 ± 0,03 | 0,22 ± 0,02 |
| **176** | 1400 | 1400 | tetradecane | 629-59-4 | Q2 | | 0,59 ± 0,11 | | |
| **177** | 1411 | 1389 | ethyl 9-decenoate | 67233-91-4 | | 0,01 ± <0,001 | | 0,05 ± <0,001 | |
| **178** | 1412 | | Unknown compound 51 | | | | | | 0,13 ± 0,03 |
| **179** | 1418 | | Unknown compound 52 | | | | | | 0,08 ± <0,001 |
| **180** | 1422 | | Unknown compound 53 | | | | | | 0,05 ± 0,01 |
| **181** | 1422 | | Unknown compound 54 (ester) | | | 0,01 ± <0,001 | | | |
| **182** | 1424 | 1421 | (*Z*)-*α*-bergamotene | 18252-46-5 | W2 | | | 0,03 ± <0,001 | 0,05 ± 0,01 |
| **183** | 1433 | 1433 | *(*E*)-*β*-caryophyllene | 87-44-5 | Q2,W1,W3 | 0,01 ± <0,001 | 0,15 ± 0,01 | 1,57 ± 0,21 | 5,37 ± 0,32 |
| **184** | 1443 | 1449 | *γ*-muurolene | 30021-74-0 | | | | 0,07 ± 0,02 | 0,25 ± 0,04 |
| **185** | 1444 | 1433 | *β*-copaene | 18252-44-3 | | | 0,08 ± 0,01 | | |
| **186** | 1446 | 1448 | ethyl (*E*)-2-decanoate | 7367-88-6 | Q1 | 0,03 ± <0,001 | | | |
| **187** | 1452 | 1451 | *(*Z*)-*β*-caryophyllene | 87-44-5 | W2,W3 | | | 0,03 ± <0,001 | 0,03 ± <0,001 |
| **188** | 1457 | 1457 | ethyl (*E*,*Z*)-2,4-decadienoate (PE) | 3025-30-7 | | 0,01 ± <0,001 | | | |
| **189** | 1460 | 1461 | *β-farnesene | 28973-97-9 | Q1,W2,W3 | 0,01 ± <0,001 | | 1,64 ± 0,05 | 4,04 ± 0,66 |
| **190** | 1462 | | Unknown compound 55 | | | | 0,11 ± 0,02 | | |
| **191** | 1464 | 1416 | *β*-funebrene | 79120-98-2 | | | | 0,03 ± 0,01 | 0,12 ± 0,01 |
| **192** | 1469 | 1477 | *α*-humulene | 6753-98-6 | W1,W2,W3 | 0,89 ± 0,22 | 0,04 ± <0,001 | 0,09 ± 0,02 | 0,3 ± 0,05 |
| **193** | 1478 | | Unknown compound 56 | | | | | | 0,06 ± <0,001 |
| **194** | 1490 | 1484 | *γ*-curcumene | 28976-68-3 | W1,W2 | | | 0,12 ± 0,01 | 0,39 ± 0,07 |
| **195** | 1495 | 1496 | *ethyl undecanoate | 627-90-7 | | 0,27 ± 0,06 | | | |
| **196** | 1496 | 1496 | germacrene-D | 23986-74-5 | W1,W2,W3 | | | 1,37 ± 0,15 | 3,3 ± 0,43 |
| **197** | 1497 | | Unknown compound 57 (alkane) | | | | 0,53 ± 0,06 | | |
| **198** | 1502 | 1495 | zingiberene | 495-60-3 | W2,W3 | | | 0,28 ± 0,02 | 0,07 ± 0,01 |
| **199** | 1511 | 1507 | **α*-farnesene | 502-61-4 | Q1,Q2,W1,W2 | 12,5 ± 1,72 | 1,83 ± 0,27 | 0,01 ± <0,001 | 0,04 ± 0,01 |
| **200** | 1511 | | Unknown compound 58 | | | | | | 0,24 ± 0,06 |
| **201** | 1520 | | Unknown compound 59 | | | | | 0,04 ± 0,01 | |
| **202** | 1528 | 1524 | *γ*-cadinene | 1460-97-5 | W3 | | | 0,03 ± <0,001 | 0,09 ± 0,01 |
| **203** | 1532 | 1525 | *β*-sesquiphellandrene | 20307-83-9 | W2 | | | 0,09 ± 0,01 | 0,08 ± 0,01 |
| **204** | 1542 | | 2,6,10-trimethyl tetradecane | 14905-56-7 | | | 0,16 ± 0,01 | 0,01 ± <0,001 | |
| **205** | 1551 | | Unknown compound 60 | | | | 0,12 ± <0,001 | 0,01 ± <0,001 | 0,02 ± 0,01 |
| **206** | 1571 | | methyl (*Z*)-5-dodecenoate | | | 0,78 ± 0,14 | | | |
| **207** | 1575 | | methyl 3,6-dodecadienate | 16106-01-07 | | 0,45 ± 0,03 | | | |
| **208** | 1579 | | Unknown compound 61 | | | 0,08 ± 0,01 | | | |

| | | | | | | | | | |
|---|---|---|---|---|---|---|---|---|---|
| **209** | 1585 | | (*Z*)-5-dodecenoic acid | 2430-94-6 | | 0,43 ± 0,09 | | | |
| **210** | 1594 | 1595 | *dodecanoic acid, ethyl ester | 106-33-2 | Q1,Q2 | 1,66 ± 0,07 | | | |
| **211** | 1598 | | Unknown compound 62 | | | | 0,15 ± 0,02 | | |
| **212** | 1600 | | Unknown compound 63 | | | 0,03 ± <0,001 | | | |
| **213** | 1602 | 1589 | caryophyllene oxide | 1139-30-6 | W1,W2,W3 | | | 0,99 ± 0,25 | 1,31 ± 0,2 |
| **214** | 1624 | | Unknown compound 64 (ester) | | | 0,22 ± 0,03 | | | |
| **215** | 1640 | | Unknown compound 65 (ester) | | | 0,01 ± <0,001 | | | |
| **216** | 1647 | | Unknown compound 66 (acid) | | | 0,05 ± 0,01 | | | |
| **217** | 1664 | | Unknown compound 67 (ester) | | | 0,08 ± 0,08 | | | |
| **218** | 1669 | | ethyl 10-undecanoate | 692-86-4 | | 0,49 ± 0,06 | | | |
| **219** | 1675 | | Unknown compound 68 | | | 0,02 ± <0,001 | | | |
| **220** | 1735 | | 1,1,3-trimethyl-3-phenyl indan | 3910-35-8 | | 0,001 ± <0,001 | | | |
| **221** | 1760 | | Unknown compound 69 | | | 0,47 ± 0,06 | | | |
| **222** | 1768 | | Unknown compound 70 (ester) | | | 0,66 ± 0,03 | | | |
| **223** | 1794 | 1794 | ethyl tetradecanoate | 0124-06-01 | | 0,01 ± <0,001 | | | |
| **224** | 1956 | | Unknown compound 71 | | | 0,02 ± <0,001 | | | |
| **225** | 2054 | | Unknown compound 72 | | | 0,001 ± <0,001 | | | |
| | | | | | | **99,536** | **99,47** | **99,82** | **99,058** |

aCalculated Kovats Retention indices using C8-C20 alkane standards (Sigma-Aldrich)

bRetention indices from NIST database that closest matched with GC-MS setup described in the experiment

*Electrophysiologically active compounds tested with synthetic standards

Q1 Tsuneya T., Ishihara M., Shiota H., Shiga M, Volatile Components of Quince Fruit (Cydonia oblonga Mill.). (1983). Agricultural and Biological Chemistry, 47(11), 2495–2502

Q2 Griñán, I., Galindo, A., Rodríguez, P., Morales, D., Corell, M., Centeno, A., J. Collado-González, A. Torrecillas, A. Carbonell-Barrachina & ernández, F. (2019). Volatile composition and sensory and quality attributes of quince (Cydonia oblonga Mill.) fruits as affected by water stress. Scientia Horticulturae, 244, 68-74.

Q3 Tateo, F., & Bononi, M. (2010). Headspace-SPME analysis of volatiles from quince whole fruits. Journal of Essential Oil Research, 22(5), 416-418.

W1 Buttery, R. G., Light, D. M., Nam, Y., Merrill, G. B., & Roitman, J. N. (2000). Volatile components of green walnut husks. Journal of Agricultural and Food Chemistry, 48(7), 2858-2861.

W2 Casado, D., Gemeno, C., Avilla, J., & Riba, M. (2008). Diurnal variation of walnut tree volatiles and electrophysiological responses in Cydia pomonella (Lepidoptera: Tortricidae). Pest Management Science: formerly Pesticide Science, 64(7), 736-747.

W3 San Román, I., Bartolomé, L., Gee, W. S., Alonso, R. M., & Beck, J. J. (2015). Comparison of ex situ volatile emissions from intact and mechanically damaged walnuts. Food Research International, 72, 198-207.

Table S2.) Synthetic compounds used in volatile profile analyses, with purity and supplier given for reference.

| Compounds | CAS | Purity and Supplier of Synthetic standards |
|---|---|---|
| ethyl isobutyrate | 97-62-1 | 99% (Sigma-Aldrick/Merck) |
| isobutyl acetate | 110-19-0 | ≥98% (Sigma-Aldrick/Merck) |
| 3-hexanal | 4440-65-7 | 50% (Alfa Chemistry) |
| ethyl butyrate | 105-54-4 | 99% (Sigma-Aldrich/Merck) |
| butyl acetate | 123-86-4 | ≥98% (Sigma-Aldrick/Merck) |
| (*E*)-3-hexen-1-ol | 928-97-2 | 97% (Sigma-Aldrich/Merck) |
| ethyl isovalerate | 108-64-5 | 98% (Sigma-Aldrich/Merck) |
| (*Z*)-3-hexen-1-ol | 928-96-1 | 98% (Sigma-Aldrich/Merck) |
| isoamyl acetate | 123-92-2 | 97% (Sigma-Aldrich/Merck) |
| 2-methylbutyl acetate | 75-65-0 | 99% (Sigma-Aldrich/Merck) |
| ethyl pentanoate | 539-82-2 | 99% (Sigma-Aldrich/Merck) |
| amyl acetate | 628-63-7 | 97% (Sigma-Aldrich/Merck) |
| *α*-pinene | 80-56-8 | 98% (Sigma-Aldrich/Merck) |
| ethyl tiglate | 5837-78-5 | 98% (Sigma-Aldrich/Merck) |
| sabinene | 3387-41-5 | 75% (Sigma-Aldrich/Merck) |
| *β*-pinene | 127-91-3 | ≥90%(Sigma-Aldrich/Merck) |
| *β*-myrcene | 123-35-3 | ≥90%(Sigma-Aldrich/Merck) |
| ethyl hexanoate | 123-66-0 | ≥99% (Sigma-Aldrick/Merck) |
| (*Z*)-3-hexenyl-acetate | 3681-71-8 | 95% (Sigma-Aldrick/Merck) |
| hexyl acetate | 142-92-7 | 99% (Sigma-Aldrick/Merck) |
| limonene | 138-86-3 | ≥90%(Sigma-Aldrich/Merck) mixture of isomers 1:1 |
| eucalyptol | 470-82-6 | 99% (Sigma-Aldrick/Merck) |
| *β*-ocimene | 502-99-8 | ≥90%(Sigma-Aldrich/Merck) mixture of isomers |
| *γ*-terpinene | 99-85-4 | 97% (Sigma-Aldrick/Merck) |
| ethyl heptanoate | 106-30-9 | ≥98% (Sigma-Aldrick/Merck) |
| linalool | 78-70-6 | 97% (Sigma-Aldrick/Merck) |
| heptyl acetate | 0112-06-01 | ≥98% (Sigma-Aldrick/Merck) |
| (*E*)-4,8-dimethyl-1,3,7-nonatriene | 19945-61-0 | 96% (BioSynth) |
| thujone | 546-80-5 | ~70% α-thujone basis, ~10% β-thujone basis (Sigma-Aldrich/Merck) |
| ethyl octanoate | 106-32-1 | ≥ 98 % (Sigma-Aldrich/Merck) |
| ethyl nonanoate | 123-29-5 | 97 % (Sigma-Aldrich/Merck) |
| ethyl decanoate | 110-38-3 | ≥ 98 % (Sigma-Aldrich/Merck) |
| (*E*)-*β*-caryophyllene | 87-44-5 | > 80% (Sigma-Aldrich/Merck) |
| (*Z*)-*β*-caryophyllene | 118-65-0 | > 80% (Sigma-Aldrich/Merck) |
| (*Z*)-*β*-farnesene | 28973-97-9 | 96% (Alfa Chemistry) |
| ethyl undecanoate | 627-90-7 | 97 % (Sigma-Aldrich/Merck) |
| *α*-farnesene | 113244-64-7 | >90 % (BioSynth) |
| ethyl dodecanoate | 106-33-2 | ≥ 99 % (Sigma-Aldrich/Merck) |
